# One size fits all? A latent profile analysis to identify care professional subgroups based on implementation determinants

**DOI:** 10.1186/s43058-025-00794-x

**Published:** 2025-11-17

**Authors:** Eveline M. Dubbeldeman, Rianne M. J. J. van der Kleij, Jessica C. Kiefte-de Jong, Hester M. Diderich, Isabelle L. L. Gerding, Matty R. Crone

**Affiliations:** 1 Department of Public Health and Primary Care, Leiden University Medical Center, Leiden, The Netherlands; 2 Department of Public Health and Primary Care/Health Campus The Hague, Leiden University Medical Center, The Hague, The Netherlands; 3 Emergency Department, Medical Center Haaglanden, The Hague, The Netherlands; 4 Department of Health Promotion, Maastricht University, Maastricht, The Netherlands

**Keywords:** Implementation determinants, Childcheck, Mental health care, Forensic care, Vulnerable children and families, Latent profile analyses

## Abstract

**Introduction:**

While the importance of a more holistic approach to implementation science, recognizing the interconnection among implementation determinants and the heterogeneity of context and care professionals (CPs), has long been acknowledged, recent research has increasingly focused on these issues. Despite this growing attention, the practical application of these insights within implementation research remains limited. In this study, we aimed to identify distinctive subgroups of CPs based on their profiles of implementation determinants concerning the Childcheck, a guideline facilitating early identification of child abuse based on parental characteristics. We also explored the influence of organization type on subgroups of CPs with specific implementation characteristics (subgroup membership) and assessed their relationship to CPs implementation level.

**Methods:**

A total of 562 Dutch CPs in Mental Health Care (aMHC) and Forensic Care settings completed a self-reported questionnaire on Childcheck implementation determinants. We conducted Latent Profile Analysis to identify subgroups of CPs. The influence of organization type on subgroup membership was examined using Chi-Squared test and we explored the impact of subgroup membership on implementation levels using a one-way ANOVA.

**Results:**

We identified five distinct subgroups. Subgroup A (Reporting Center for Child Abuse and Neglect (RCCAN) collaboration issues, 11.7%) faced issues related to the external organization, such as feedback and collaboration issues. Subgroup B (RCCAN collaboration and organizational issues, 5.0%) encountered challenges with both the external and internal organization, including issues with financial resources and formal agreements, resulting in the lowest implementation level. Subgroup C (Limited implementation issues, 9.4%) demonstrated relatively high ratings across determinants, achieving the highest implementation level. CPs in subgroup D (CP-client interaction issues, 37.7%) encountered challenges in CP-client interaction. CPs in subgroup E (Indifferent attitudes towards implementation, 36.1%) expressed low to average retings, were mainly from aMHC settings, and reported a low to average implementation level.

**Conclusions:**

This study highlights the importance of tailored implementation plans to address each subgroup's specific needs and challenges, instead of employing a one-size-fits-all approach. Latent Profile Analysis successfully revealed the variations in implementation determinants among CPs in aMHC and Forensic Care settings. Tailoring implementation strategies for these subgroups is key to successful guideline implementation and enhancing the well-being of vulnerable children and families.

**Supplementary Information:**

The online version contains supplementary material available at 10.1186/s43058-025-00794-x.

Contributions to the literature

- Recent implementation research highlights the growing emphasis on a holistic approach, recognizing the complex interconnection among determinants and the heterogeneity of care professionals, though practical application remains limited.
- This study shows that Latent Profile Analysis is a valuable method to identify subgroups of professionals, each characterized by unique implementation determinants, allowing for the development of tailored implementation strategies.
- Addressing the specific needs of these subgroups can improve guideline implementation and enhance the well-being of vulnerable children and families.

## Introduction

In recent years, interest in implementation research within child and family care settings has grown, particularly in determinants influencing the implementation of guidelines and interventions [1–5]. While many studies have focused on the influence of individual determinants—such as resource availability, self-efficacy, and guideline complexity—there is increasing recognition that implementation success is rarely determined by single factors in isolation. Instead, it depends on how these determinants are interconnected (i.e., the way in which determinants tend to co-occur and form meaningful patterns across implementation contexts) and how they influence implementation processes [6–10]. This has led to calls for a more holistic approach, viewing context as a multi-level, dynamic, and interconnected system of influences. As described by Nilsen and Bernhardsson (2019), determinants exist at the micro level (e.g., professionals), meso level (e.g., teams, departments), and macro level (e.g., policy, funding), and emphasizes that these factors are not independent but interconnected. For example, while lack of time is often perceived as a barrier to implementation, it can result from various factors such as, insufficient leadership, low service priority, or inappropriate workflow. Effectively addressing the issue of time constraints in an implementation program requires targeted strategies tailored to the identified causes, rather than attempting to address the barrier in isolation [7]. Additionally, Care Professionals (CPs) and care settings can differ in terms of resources, organizational culture, professional expertise, and patient populations. This contextual heterogeneity implies that CPs operate under markedly different conditions, shaping the barriers and facilitators they encounter, making standardized implementation strategies inadequate. This underscores the need for tailored approaches that are responsive to specific contextual situations [11, 12].

Several theoretical frameworks support this shift toward contextualized and person-centered implementation research. The Consolidated Framework for Implementation Research (CFIR) categorizes determinants into contextual domains, such as the outer and inner settings, individual characteristics, and implementation processes, and emphasizes their interconnection [13]. Likewise, Rogers’ Diffusion of Innovations theory [14] and Greenhalgh’s model [15] emphasize that adoption processes are shaped by cultural, professional, and organizational contexts. Together, these frameworks suggest that implementation success hinges on how contextual determinants co-occur and form meaningful patterns across settings and CPs. Such patterns may indicate distinct subgroups of CPs who share similar configurations of determinants. Identifying these subgroups enables researchers and policymakers to understand the unique needs and challenges of different CPs, allowing for the development of tailored strategies for each subgroup, increasing the likelihood of successful implementation. This subgroup-focused approach also improves resource allocation by directing implementation efforts where they are most needed, ensuring efficient use and maximizing their impact. Despite these benefits, current literature has paid limited attention to quantitatively identifying specific subgroups based on implementation determinants. While previous qualitative research has revealed some patterns and trends [16–18], a systematic quantitative approach to subgroup identification remains largely unexplored.

This study presents a method to systematically capture contextual variation—or heterogeneity—in implementation determinants among care CPs. To this end, we conducted Latent Profile Analysis (LPA), a person-centered statistical technique that identifies subgroups of individuals based on shared patterns across multiple determinants [19]. Unlike variable-centered techniques such as Confirmatory Factor Analysis or Principal Component Analysis, LPA examines how determinants co-occur within individuals, allowing for a nuanced understanding of how they cluster in practice. This approach facilitates the identification of distinct profiles of CPs, each characterized by unique configurations of determinants, thereby supporting the development of tailored implementation strategies.

We used data collected during the implementation of the Childcheck, a guideline designed to support the early identification of child abuse based on parental risk factors, such as domestic violence, substance abuse, and suicide attempt or other severe psychiatric problems [20, 21]. The Childcheck is part of the mandatory Model Protocol for Domestic Violence and Child Abuse and applied to all professionals working with adult patients or children. This protocol outlines five steps: 1) Identify signs; 2) Consult a colleague and, if necessary, with the Reporting and Advice Center for Domestic Violence and Child Abuse (RCCAN; in Dutch: *Veilig Thuis*); 3) Talk to the person(s) involved; 4) Assess whether domestic violence or child abuse has occurred. If in doubt, consult with the RCCAN; 5) Decide to offer help or report to the RCCAN. The Child Check is conducted at step one, focusing on parental indicators rather than child-specific symptomsIt demonstrated strong predictive validity: child abuse was confirmed in 91% of the cases, with other forms of abuse identified in 7% of the cases, supporting its effectiveness as a screening tool [20]. In 2018, the Dutch Ministry of Health, Welfare, and Sport initiated a Childcheck implementation impulse—a nationally coordinated initiative aimed at accelerating the translation of policy into practice. The impuse involved targeted support, monitoring, and evaluation activities to strengthen the implementation of the Childcheck inMental Health Care (aMHC) and Forensic Care (FC). These settings involve individuals facing complex mental health issues, co-occurring disorders, or past trauma and legal involvement all of which may endanger their children's safety [20]. Improved Childcheck implementation in these contexts facilitates early risk detection and intervention, reinforcing CPs’ duty to safeguard all parties and incorporate a family perspective.

One of the steps in the implementation impulse was to identify determinants influencing the implementation of the Childcheck. Using data from the implementation of the Childcheck within aMHC and FC, we aimed to:

- 1) Identify subgroups of CPs working in aMHC and FC based on their perceptions regarding the implementation of the Childcheck.
- 2a) Explore whether subgroup allocation is related to organization type.
- 2b) Assess how subgroup allocation is related to CPs’ implementation level.

## Methods

For this study, we conducted retrospective, cross-sectional analyses to identify subgroups among CPs based on data concerning determinants influencing the Childcheck implementation. The implementation impulses initiated by the ministry were translated into implementation programs by a team of professionals, including researchers, domestic violence and child abuse policy officers, and statisticians. Additionally, an advisory group, consisting of policymakers and professionals from various aMHC and FC settings, played a key role by offering insights, feedback, and advice on the implementation programs. The Haaglanden Medical Center (The Hague, The Netherlands) was responsible for executing the implementation impulses across The Netherlands. The Medical Ethics Committee at Leiden University Medical Center reviewed our research proposal (proposal number WSC-2022–38), and determined that the Dutch Medical Research Involving Human Subjects Act did not apply. Our reporting adheres to the STROBE guidelines (Appendix A) [22].

### Participants and procedures

Two distinct implementation programs were developed: one for CPs in Dutch aMHC and another for FC organizations. The aMHC implementation program expanded on a previous three-year implementation program (2016–2018), involving 103 organizations. By the end of this program, 37 organizations had not yet implemented the Childcheck or failed to meet the pre-implementation requirements, such as adequate ICT infrastructure and the assignment of a policy officer. These organizations were invited to participate in an additional implementation program in 2019. Some organizations that participated in the initial implementation program were unable to join the extended program due to mergers, though ten ultimately participated. Additionally, we recruited other aMHC organizations that had had not previously participated in the initial program, of which two agreed to join. In total, twelve aMHC organizations participated. In the Netherlands, aMHC services encompasses the diagnosis, treatment, and support of individuals and their family dealing with various mental health issues, such as depression, anxiety, and psychiatric disorders. Funding for aMHC is commonly sourced from the Health Insurance Act.

The FC implementation program started in 2020, involving the recruitment of Forensic MHC, Probation Service, and the Salvation Army. The FC aims to safely reintegrate offenders into society, recognizing that punishment alone is insufficient for those with mental disorders, intellectual disabilities, or addiction. Specifically, forensic MHC focuses on treating mental health issues related to criminal behavior, while Probation Service offers guidance for post-sentence reintegration and aims to prevent recidivism. The Salvation Army offers various support services, including assistance for the homeless, substance abusers, and the socially vulnerable. In the Netherlands, government funding supports Forensic MHC and Probation Services, while The Salvation Army’s financing depends on the type of care, with funding sources including the municipality, government, or the Health Insurance Act. A total of 32 FC organizations decided to participate (i.e., 19 Forensic MHC, 10 Probation Services, and 3 the Salvation Army). In total, 44 aMHC and FC organizations joined the implementation programs (Appendix B).

Communication was facilitated through organizational representatives. Each representative distributed a questionnaire among CPs via mail, providing a link to an online platform (https://kindcheck-ggz.nl and https://kindcheck-forensisch.nl). Reminders were sent to the representatives to ensure the distribution of the questionnaires. In aMHC organizations, questionnaires were distributed from February 2019 to November 2020, and in FC organisations, from October 2020 to June 2022. The implementation programs were initially not designed with a research focus, butwere intended to evaluate, assist, and monitor the implementation of Childcheck. As a result, individual informed consent was not obtained. However, the questionnaires were fully anonymous, with only the organization name and department being recorded. CPs were informed via the website about the implementation program, and that data would be collected anonymously and shared with their respective organizations to optimize implementation of the Childcheck.

### Questionnaire

We developed a questionnaire (Appendix C) to assess CPs’ perceptions on determinants influencing Childcheck implementation. This questionnaire is based on the theoretical and evidence-based Measurement Instrument for Determinants of Innovations (MIDI) framework, developed by Fleuren et al. [23], which measures determinants affecting the implementation of innovations. The MIDI includes four categories:determinants related to the user (e.g., knowledge), the innovation (e.g., procedural clarity), the organization (e.g., financial resources) and the socio-political context (e.g., law and regulations). For the development of our questionnaire, we focused on the first three categories. Determinants relating to the socio-political context were not included, as these could not be directlyinfluenced by the organization or CP. Additionally, we deductively included items derived from evaluations with the project team and the advisory group. Additionally, we included questions to assess the extent to which CPs adhere to the recommendations within the Childcheck (i.e., implementation level), with four items such as ‘Do you apply the Childcheck in the initial client meeting?’.

The final questionnaire consisted of 37 questions organized into four categories: 1) the user (the CP, twenty items), 2) the innovation (Childcheck, four items), 3) the organization (aMHC and FC organizations, nine items), and 4) the implementation level (four items). Items related to implementation determinants were rated using a 5-point Likert scale (1—totally disagree to 5—totally agree), with an additional ‘not applicable/I don't know’ option. Implementation level items were rated on a 4-point Likert scale (1 – never to 4—always), except for one item, which useda 5-point scale (1—totally disagree to 5—totally agree). Prior to distribution, we conducted a review for clarity and time estimation. The total time needed to fill out the questionnaire was approximately 20–30 min.

### Statistical analyses

#### Data cleaning

We imported data to IBM SPSS Statistics 25 for Windows. Since all questions were mandatory, there were no missing values. The category ‘not applicable/I don’t know’ was recoded as 3 (neutral), and we adjusted the categories of the reverse-worded items. We excluded data from CPs who were not familiar with the Childcheck. The cleaned data was imported into RStudio version 4.3.1 for further analysis.

#### Reliability and scaling

Several determinants in our study were measured using multiple items, which together reflect a construct representing the underlying determinant. We applied Item Response Theory (IRT) using the ‘mirt’ package to analyze the item characteristics and assess how the items within each construct contribute to the measurement of these underlying determinants (Appendix D) [24, 25]. Specifically, we used the Generalized Partial Credit Model (GPCM) of IRT to evaluate the following implementation determinants: coordinator, partnership and connection, client cooperation, descriptive norm, knowledge, professional obligation, outcome expectations, and implementation level. The GPCM allows for the assessment of both the item discrimination (i.e., *a-*parameter, which measures an item's ability to distinguish between different scores of the determinant being measured) and threshold parameter (i.e., *b*-parameter, which identifies the score of the determinant at whichthe choice of response category changes). Items with a-values above one and well-distributedb-parameters, were considered suitable for representing the construct. Item information scores further supported reliability and item contribution. We calculated weighted person scores for each participant based on item discrimination and threshold parameter, offering a refined estimate of the underlying determinant. These scores were rescaled to a 1–5 range to ensure alignment across all measured determinants.

Additionally, we conducted a Confirmatory Factor Analysis (CFA) to further assess the validity of the constructs based on the IRT analyses; one model for all determinants and another for the implementation level. CFA tests whether the observed data fit the hypothesized model structure, confirming that the constructs were measured as intended. We evaluated model fit using indices like CFI, TLI, RMSEA, and SRMR [26]. A good fit indicates that the constructs are well-defined and measured appropriately by the related items. Finally, we calculated Composite Reliability to assess the internal consistency, ensuring that the items within each construct consistently measure the same underlying determinant, while accounting for potential variations in item loadings (Appendix E) [27].

#### Subgroup identification

We conducted a LPA using the ‘tidyLPA’ package (Appendix F) [28]. LPA is a technique used to identify unobserved (latent) subgroups or profiles within a heterogenic population by examining patterns in observed variables [19]. Unlike techniques such as distance-based clustering methods like k-means, LPA focuses on identifying underlying patterns and structures in the data, assuming that the subgroups represent different distributions of the data. We applied both Model 1 and Model 3 LPAs with one to ten profiles. Model 1 assumed equal variances across profiles and zero covariances between indicators, while Model 3 assumed equal variances and equal covariances across profiles. Model fit was assessed using the ‘Mclust’ package [29], with selectionbased on fit indices and interpretability [30]. To compare the relative fit among competing models, we used the Integrated Completed Likelihood (ICL) with higher values indicating a better fit to the data, as well as the Akaike Information Criterion (AIC) and Bayesian Information Criterion (BIC), with lower values indicating a better fit. It is important to note that AIC values may continuously decrease with large sample sizes, and a high BIC could suggest an overly complex model [31]. Additionally, we assessed the models’ entropy, which indicates the clarity of profile distinction, with values ranging from 0 to 1 (with higher values indicating better distinction) [32]. Profiles were interpreted by examining the mean ratings of implementation determinants within each profile, revealing unique subgroup characteristics and their practical relevance to Childcheck implementation. ANOVA, including Tukey's post-hoc analyses identified significant differences in CPs’ perceived implementation determinant between subgroups.

#### Relationship with organization type and implementation level

To examine whether subgroup membership differs across organization type (i.e., aMHC, Forensic MHC, Probation Services, and the Salvation Army), we performed a chi-square test. To account for multiple comparisons, we applied Bonferroni corrections to control for Type I errors. Our hypothesis was that organization type would be associated with group membership (significant level was set at α = 0.05 and α = 0.0025 with Bonferroni correction). We also explored differences in implementation level across subgroups using a one-way ANOVA with a significance level set at α = 0.05. Post-hoc pairwise comparisons were conducted using Tukey’s Honestly Significant Difference method to identify specific group differences when the ANOVA showed significance. Our hypothesis was that there would be significant differences in implementation levels among the identified subgroups.

## Results

In total, 603 CPs completed the questionnaire (aMHC: 204, forensic MHC: 198, Probation Service: 161, and the Salvation Army: 40). Forty-one cases were excluded due to unfamiliarity with the Childcheck, resulting in a final dataset of 562 cases for the subsequent analysis (aMHC: 193, forensic MHC: 180, Probation Service: 159, and the Salvation Army: 30).

### Reliability and scaling

Based on the IRT analyses, we formed seven reliable construct, each effectively representing the following determinants: coordinator (two items), partnership and connections (four items), client cooperation RCCAN (two items), descriptive norm (two items), knowledge (two items), professional obligation (four items), and outcome expectations (two items). The construct implementation level was formed by three items; the item (‘I conduct the Childcheck in accordance with step 1 of the Reporting Code’) was not included in the construct due to poor discrimination, inconsistent location parameters, poor information, and poor fit to the model (Appendix D).

The CFA confirmed a good fit for the model with determinants, with the CFI (0.960) and TLI (0.946) exceeding the commonly accepted threshold of 0.95, indicating a strong fit. The RMSEA (0.050) and SRMR (0.039) also support good model adequacy, as both values are within acceptable limits (RMSEA < 0.06 and SRMR < 0.08). The factor loadings for the determinants ranged from 0.548 to 0.894, with most items showing factor loadings above 0.6, indicating acceptable construct representation. However, a few items, particularly in Professional Obligation and Coordinator, had factor loadings below 0.6. Additionally, Composite Reliability values range from 0.756 to 0.889, indicating good internal consistency for all constructs. The CFA for implementation level showed perfect model fit, likely due to the small number of items. The factor loadings ranged from 0.651 to 0.796, and the Composite Reliability was 0.795, indicating good internal consistency (Appendix E).

In total, 22 determinants divided over three domains were used in the LPA: the innovation (compatibility, observability, procedural clarity, relative priority), the organization (access to knowledge, coordinator, financial resources, formal ratification, partnership and connection, time, client cooperation Childcheck, client cooperation RCCAN), and the professional (communication skills, descriptive norm, general skills, routine, implementation needs, knowledge, outcome expectations, professional obligation, relationship with client, social support).

### Subgroup identification

We assessed the fit indices of various models, ultimately selecting the model that best represented distinctive and interpretable subgroups within the data (Appendix F). Overall, Model 3 showed the best performance on AIC and BIC, indicating strong statistical fit, while Model 1 yielded better results on ICL and Entropy, suggesting higher classification certainty and a clearer profile structure. The lowest AIC value was observed for Model 3 with 10 profiles, followed by Model 3 with 8 profiles. In terms of BIC, Model 3 with 2 profiles performed best, with Model 1 with 9 profiles ranking second. The highest ICL was also found in Model 3 with 2 profiles, followed by Model 1 with 8 profiles. Regarding classification certainty, the highest Entropy was observed in Model 3 with 2 profiles, followed by Model 1 with 5 profiles. Although some competing models showed better statistical fit, the five-profile solution from Model 1 was selected based on its stronger performance on ICL and Entropy, as well as the clearer and more interpretable profile structure. This model offered the best overall balance between fit indices and classification certainty, making it the most suitable choice for identifying meaningful subgroups in the data. Table 1 provides estimates for all implementation determinants in model 1 with 5 profiles (i.e., subgroups of CPs), and a visual representation of the subgroups is shown in Appendix F. The ANOVA shows significant differences across all determinants, confirming the presence of distinct subgroups. The post-hoc analyses further support this by showing that nearly all pairwise comparisons are significantly different (Appendix F). This reinforces the validity of the LPA subgroups and confirms that they are meaningfully distinct in terms of CPs’ perceived implementation determinants.

**Table 1:** Mean ratings of implementation determinants in the five identified subgroups and the total group

Determinant		RCCAN collaboration issues	RCCAN collaboration and organizational issues	Limited implementation issues	CP-client interaction issues	Indifferent attitudes towards implementation	ANOVA	Total
		*n* = 66, 11.7%	*n* = 28, 5.0%	*n* = 53, 9.4%	*n* = 212, 37.7%	*n* = 203, 36.1%	F-value	*n* = 562
Innovation	Compatibility	4.21	**2.56**	3.86	*4.23*	3.21	57.922***	3.75
	Observability	**1.19**	**1.19**	*3.74*	3.33	2.85	170.302***	2.83
	Procedural clarity	4.14	**2.94**	3.36	*4.45*	3.47	45.630***	3.88
	Relative priority	3.20	*4.18*	3.34	**2.39**	2.84	29.135***	2.83
Organization	Access to knowledge	3.96	**2.56**	3.96	*4.04*	2.95	47.662***	3.56
	Coordinator	2.87	**2.09**	3.21	*3.63*	2.90	20.017***	3.16
	Financial resources	3.31	**1.34**	3.80	*3.85*	3.06	36.015***	3.37
	Formal ratification	4.18	**1.98**	3.99	*4.35*	3.17	71.434***	3.76
	Partnership & connections	**1.96**	**1.76**	3.73	*3.74*	3.04	94.892***	3.17
	Time	4.01	**2.10**	*4.30*	4.11	2.96	70.760***	3.61
Professional	Client cooperation Childcheck	3.33	*4.59*	3.56	**2.31**	2.80	59.028***	2.85
	Client cooperation RCCAN	4.27	*4.51*	**2.59**	2.89	3.25	47.725***	3.24
	Communication skills	2.79	*3.68*	3.60	**2.31**	2.58	30.230***	2.66
	Descriptive norm	*3.94*	3.15	4.16	3.66	**3.05**	31.794***	3.50
	General skills	4.16	**3.12**	3.58	*4.46*	3.54	40.719***	3.94
	Implementation needs	**2.16**	3.11	2.72	**2.28**	*3.27*	35.298***	2.70
	Knowledge	3.85	**2.23**	3.21	*4.23*	2.94	90.140***	3.53
	Outcome expectations	**2.26**	**1.88**	*3.60*	3.35	2.85	35.298***	2.99
	Professional obligation	3.76	**3.10**	3.14	*4.20*	4.08	31.312***	3.95
	Relationship client	3.03	*3.94*	3.93	**2.32**	2.68	49.702***	2.77
	Routine	3.85	**1.49**	3.60	*3.92*	2.55	102.224***	3.27
	Social support	3.90	**1.52**	3.94	*4.32*	3.32	66.272***	3.73

Bold values represent the lowest value for each determinant or a value lower than 2.50: *Italic* values represent the highest value for each determinant

*RCCAN* Reporting Center for Child Abuse and Neglect, *CP* Care Professional

*** = p<0.001

Subgroup A (*n* = 66, 11.7%), was characterized by low mean ratings on determinants relating to the RCCAN. CPs in this profile expressed low confidence in and perceived low client satisfaction with the assistance offered by the RCCAN (2.26 and 1.19, respectively). They also scored low in determinants such as collaboration and communication with RCCAN (1.96). However, the Childcheck was compatible with CPs’ current practices (4.21), formal agreements concerning the implementation of the Childcheck were formulated by their organization (4.18), and CPs were provided with enough time (4.01) and had access to knowledge (3.96). CPs also considered clients as cooperative concerning conversations about RCCAN (4.27). Subgroup A was labeled as ‘RCCAN collaboration issues’.

Subgroup B (*n* = 28, 5.0%) shared similarities with subgroup A with low mean ratings on determinants related to the RCCAN. Additionally, profile B was marked by low mean ratings on determinants associated with the internal organization, including concerns about formal agreements (1.98) and the presence of various resources (coordinator = 2.09, financial resources = 1.34, and time = 2.10). Routine and support from colleagues or supervisors was also rated as low (1.49 and 1.52, respectively). However, CPs in this profile rated determinants related to client cooperation as high (4.59 and 4.51) and indicated that applying the Childcheck did not interfere with other activities (4.18). We labeled subgroup B as ‘RCCAN collaboration and Organizational Issues’.

Subgroup C (*n* = 53, 9.4%) was characterized by overall average to high ratings, except for client cooperation concerning conversations with RCCAN (2.59). CPs in this profile reported to be provided with sufficient time (4.30) and perceived that the Childcheck was applied by their colleagues (4.16). Subgroup C was labeled as ‘Limited implementation issues’.

Subgroup D (*n* = 212, 37.7%), was characterized by overall high ratings on the determinants. However, CPs in this profile encountered issues when integrating the Childcheck into practice. They more often reported that applying the Childcheck interfered with their other activities (2.39), that they lacked communication skills (2.31), and were concerned that applying the Childcheck might harm their relationship with clients (2.32). Additionally, they considered clients as poorly cooperative concerning the Childcheck (2.31). We labeled subgroup D as ‘CP-client interaction issues.

Subgroup E (*n* = 203, 36.1%), was characterized by overall low to average ratings and was accordingly labeled as ‘Indifferent attitudes towards implementation’. In this subgroup, CPs did not express particularly positive or negative opinions regarding the Childcheck or its implementation determinants.

### Relationship with organization type and implementation level

#### Organization

**Fig. 1:**
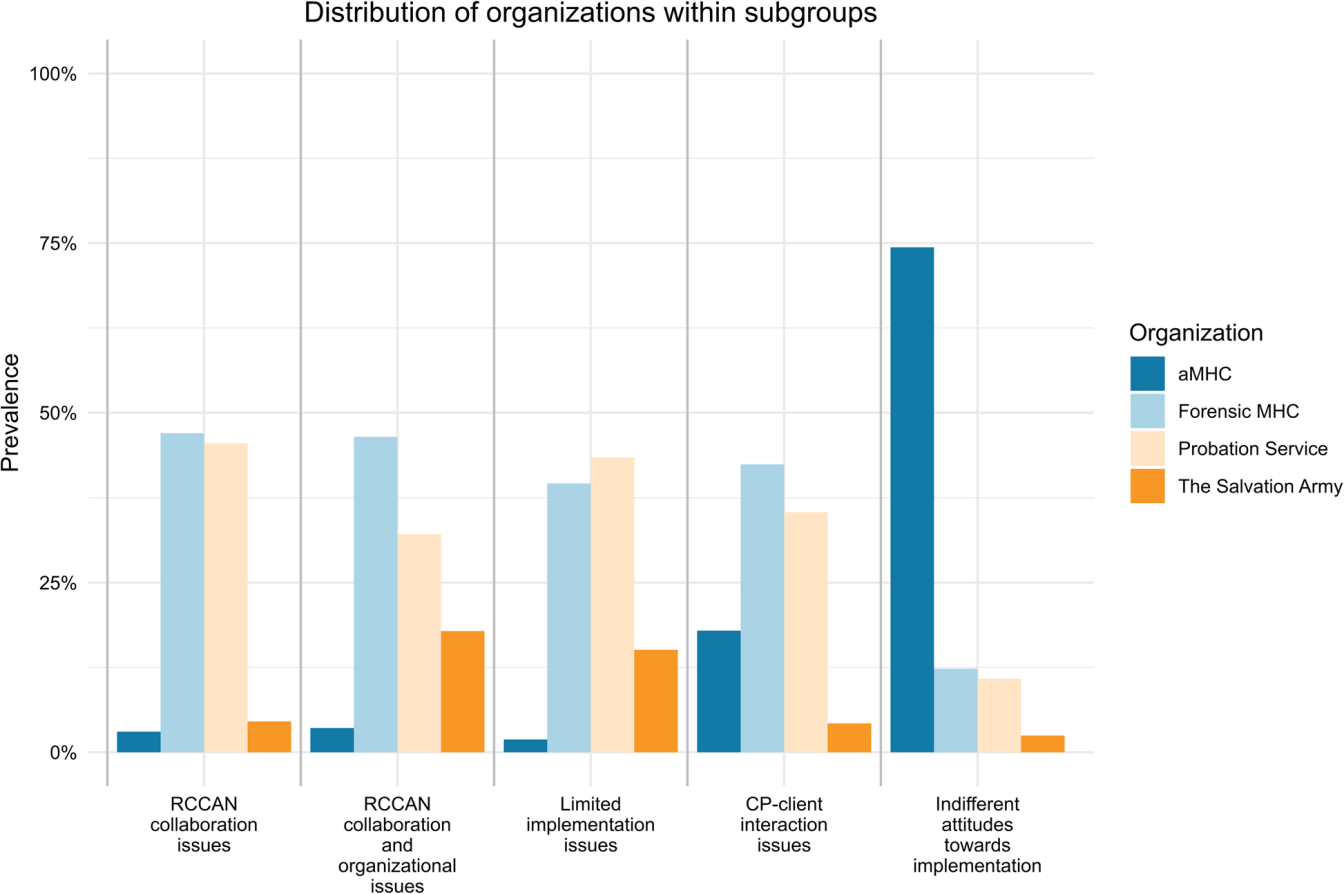
Distribution of organizations within the different subgroups. aMHC = Mental Health Care; RCCAN = Reporting Center for Child Abuse and Neglect; CP = Care Professional

The distribution of organizations within subgroups is shown in Fig. 1 and the results of the chi-square test with Bonferroni corrections are presented in Table 2. The results show a significant association between organization type and group membership among CPs (χ^2^(12, *n* = 562) = 263.09, *p* < 0.001). More specifically, CPs within aMHC were found to be more often present in the ‘Indifferent attitudes towards implementation’ subgroup (Std. Res = 15.4, *p* < 0.001), and less often to be present in the other subgroups (Limited implementation issues: Std. Res = −5.4, *p* < 0.001; CP-client interaction issues: Std. Res = −6.6, *p* < 0.001; RCCAN collaboration issues: Std. Res = −5.9, *p* < 0.001; RCCAN collaboration and organizational issues: Std. Res = −3.5, *p* < 0.05). CPs within Forensic MHC were more often present in the’CP-client interaction issues’ subgroup (Std. Res = 4.1, *p* < 0.001) and less often present in the ‘Indifferent attitudes towards implementation’ subgroup (Std. Res = −7.5, *p* < 0.001). While CPs within Probation Service were more often present in the’CP-client interaction issues’ subgroup (Std. Res = 3.1, *p* < 0.05) and ‘RCCAN collaboration issues’ (Std. Res = 3.4, *p* < 0.05), they were less often present in the ‘Indifferent attitudes towards implementation’ subgroup (Std. Res = −7.1, *p* < 0.001). CPs within The Salvation Army were more often present in the ‘Limited implementation issues’ subgroup (Std. Res = 3.4, *p* < 0.05).

**Table 2:** Chi-square tests for subgroup membership and organizations with Bonferroni correction

		**Subgroups**					
**Org**		**RCCAN collaboration issues**	**RCCAN collaboration and organizational issues**	**Limited implementation issues**	**CP-client interaction issues**	**Indifferent attitudes towards implementation**	**Total**
**aMHC**	Obs	2	1	1	38	151	193
	Exp	22.7	9.6	18.2	72.8	69.7	
	Res	−4.3	−4.3	−4.3	−4.3	−4.3	
	Std. Res	**−5.7**	**−3.5**	**−5.2**	**−6.4**	**15.0**	
**Forensic MHC**	Obs	31	13	21	90	25	180
	Exp	21.1	9.0	17.0	67.9	65.0	
	Res	2.1	2.1	2.1	2.1	2.1	
	Std. Res	2.8	1.7	1.2	**4.1**	**−7.5**	
**Probation Service**	Obs	30	9	23	75	22	159
	Exp	18.7	7.9	15.0	60.0	57.4	
	Res	2.6	2.6	2.6	2.6	2.6	
	Std. Res	**3.3**	0.5	2.6	2.9	**−6.9**	
**The Salvation Army**	Obs	3	5	8	9	5	30
	Exp	3.5	1.5	2.8	11.3	10.8	
	Res	−0.3	−0.3	−0.3	−0.3	−0.3	
	Std. Res	−0.3	3.0	**3.3**	−0.9	−2.3	
**Total**		66	28	53	212	203	562

Standardized residuals in bold are those that exceeded ± 3.0 (*p* < 0.0025)

*aMHC* Adult Mental Health Care, *RCCAN* Reporting Center for Child Abuse and Neglect, *CP* Care Professional

#### Implementation level

**Fig. 2:**
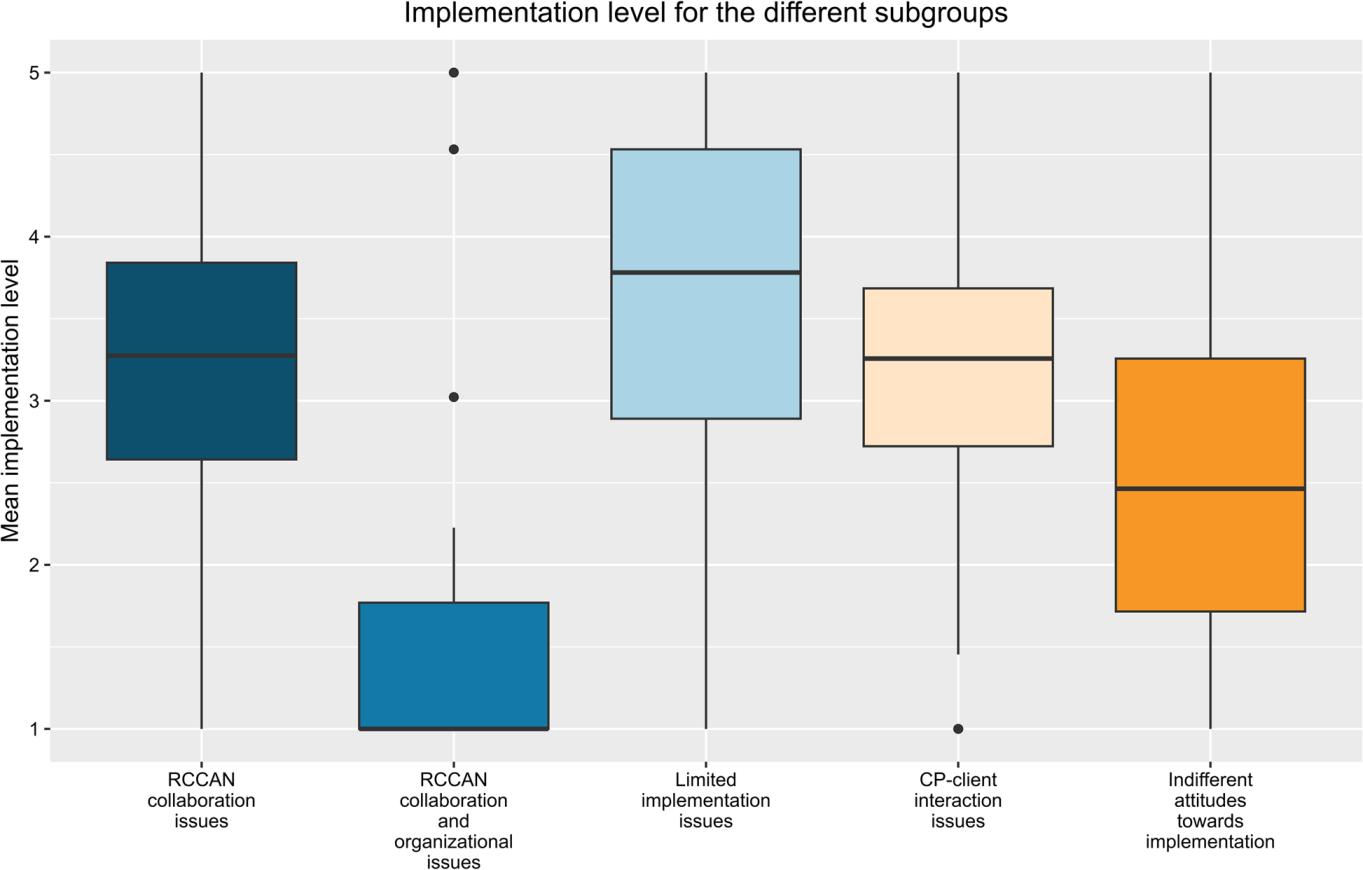
Boxplot relating to the mean implementation level for different subgroups; aMHC = Mental Health Care; RCCAN = Reporting Center for Child Abuse and Neglect; CP = Care Professional

Overall mean implementation level was 2.94 [1.00–5.00] (median = 3.04; IQR = 2.21–3.67]. Boxplots for implementation levels for each subgroup are shown in Fig. 2 and the results of the ANOVA and pairwise comparisons are presented in Table 3. The ANOVA revealed a significant overall association between subgroup membership and implementation level (F[4, >557] = 37.4, *p* < 0.001). Post hoc tests showed a significant lower mean implementation level in the ‘RCCAN collaboration and organizational issues’ subgroup compared to all other subgroups (Mean Diff. = −1.58 (RCCAN collaboration issues), −2.02 (Limited implementation issues), −1.58 (CP-client interaction issues), and −0.83 (Indifferent attitudes towards implementation), all *p* < 0.001). CPs in the ‘Limited implementation issues' subgroup had a significant higher mean implementation level compared to the CPs in the ‘RCCAN collaboration and organizational issues’ (Mean Diff. = 2.02, *p* < 0.05), ‘CP-client interaction issues’ (Mean Diff. = −0.44, *p* < 0.05), and the ‘Indifferent attitudes towards implementation’ subgroup (Mean Diff. = 1.19, *p* < 0.05).

**Table 3:** One-way ANOVA for subgroup membership and implementation level with pairwise comparison

**Source**	**Sum of squares**		**Df**	**Mean Sq**		**F-value**		***p*-value**	
Class	140.63		4	35.16		37.21		< 0.001	
Residual	526.25		557	0.94					
**Post Hoc Tukey’s HSD**									
					**Mean Diff**	**95% CI**			**Adjusted**
**Profile 1**		**Profile 2**			**Profile 2–1**	**Lower**	**Upper**		***p*-value**
RCCAN collaboration issues		RCCAN collaboration and organizational issues			−1.58	−2.17	−0.97		< 0.001
		Limited implementation issues			0.44	−0.04	0.94		0.09
		CP-client interaction issues			0.00	−0.37	0.38		1.00
		Indifferent attitudes towards implementation			−0.75	−1.12	−0.37		< 0.001
RCCAN collaboration and organizational issues		Limited implementation issues			2.02	1.40	2.64		< 0.001
		CP-client interaction issues			1.58	1.05	2.12		< 0.001
		Indifferent attitudes towards implementation			0.83	0.29	1.37		< 0.001
Limited implementation issues		CP-client interaction issues			−0.44	−0.85	−0.03		< 0.05
		Indifferent attitudes towards implementation			−1.19	−1.60	−0.78		< 0.001
CP-client interaction issues		Indifferent attitudes towards implementation			−0.75	−1.01	−0.49		< 0.001

*95% CI* 95% Confidence Intervals, *RCCAN* Reporting Center for Child Abuse and Neglect, *CP* Care Professional

## Discussion

This study aimed to identify subgroups of CPs based on perceived determinants influencing the implementation of the Childcheck within aMHC and FC. Additionally, we examined associations between subgroup membership and differences in implementation level. Five distinct subgroups were identified, each reflecting a unique profile of implementation determinants (Textbox). Subgroup A (RCCAN collaboration issues) had low mean ratings in determinants related to the RCCAN, such as collaboration, communication, and client assistance. Subgroup B (RCCAN collaboration and organizational issues) was similar to Subgroup A but had additional low ratings for internal organization determinants, like formal agreements and various resources. CPs in Subgroup B also showed low mean ratings for routine, suggesting that performing the Childcheck has not become a regular practice for them. Subgroup C (Limited implementation issues) exhibited overall relative average to high ratings. CPs in subgroup D (CP-client interaction issues) faced some difficulties integrating the Childcheck into practice, including a lack of communication skills and concerns about client relationships. CPs in subgroup E (Indifferent attitudes towards implementation) expressed average opinions, neither strongly positive nor negative. Most CPs were classified into the ‘CP-client interaction issue’ subgroup, followed by the ‘Indifferent attitudes towards implementation’ subgroup. This latter subgroup was predominantly represented by CPs working in aMHC settings. The 'Limited implementation issues' subgroup demonstrated the highest level of implementation, while the 'RCCAN collaboration and organizational issues' subgroup exhibited the lowest implementation level.

**Table:** 

**Textbox: Overview of Key Findings per Subgroup**
**A: RCCAN Collaboration Issues (n=66, 11.7%)** · Low scores on RCCAN collaboration, communication, and client support. · Childcheck aligns with workflow. · Sufficient time and knowledge, cooperative clients. · Implementation level = 3.25 **B: RCCAN Collaboration and Organizational Issues (n=28, 5.0%)** · Low scores on RCCAN collaboration and internal organization (formal agreements, resources, support). · Cooperative clients, Childcheck does not hinder work. · Implementation level = 1.67 **C: Limited Implementation Issues (n=53, 9.4%)** · Generally positive scores on all determinants. · Sufficient time, colleagues apply Childcheck. · Low client cooperation in RCCAN discussions. · Implementation level = 3.39 **D: CP-Client Interaction Issues (n=212, 37.7%)** · High scores on determinants but difficulties with client interaction. · Lack of communication skills. · Fear of harming client relationships, low client cooperation. · Implementation level = 3.25 **E: Indifferent Attitudes Towards Implementation (n=203, 36.1%)** · Low to average scores across all determinants. · No strong opinions on Childcheck or its implementation. · Implementation level = 2.50

The identified subgroups closely align with the context-specific domains of the CFIR [13], highlighting the consistency between practical outcomes and the framework’s foundational concepts. Specifically, the 'RCCAN collaboration issues' subgroup aligns with the outer setting domain, reflecting challenges related to partnerships, connections, and local attitudes. The 'RCCAN collaboration and organizational issues' subgroup spans both the outer and inner settings, emphasizing additional internal barriers, such as funding and access to knowledge. The 'CP-client interaction issues' correspond to the individual domain, including capabilities, needs, and behaviors of CPs and clients. Moreover, contextual barriers across CFIR domains appear to shape how CPs implement the Childcheck. CPs with lower implementation levels encounter more outer and inner context barriers, such as limited collaboration and insufficient organizational support. In contrast, CPs with higher implementation levels primarily face challenges within the individual domain, reflecting personal experiences, competences, and behaviors characteristic of frequent, more experienced Childcheck users. These differences in implementation level also align with Rogers’ Diffusion of Innovations theory [14]. The ‘RCCAN Collaboration and Organizational Issues’ subgroup reflects laggards, hindered by persistent systemic barriers, while the ‘Limited Implementation Issues’ subgroup resembles innovators, actively engaging with the Childcheck. Subgroup-specific profiles suggest that common implementation strategies may not be equally effective. For example, audit and feedback, which targets individual behavior, could support the ‘CP-Client Interaction Issues’ subgroup, who actively use Childcheck but face personal-level challenges [33]. For the ‘RCCAN Collaboration and Organizational Issues’ subgroup, such strategies may be less actionable unless structural conditions are first addressed. Tailoring approaches to each subgroup’s dominant barriers is key to sustainable implementation. These insights highlight the practical relevance of this study for implementing the Childcheck in aMHC and FC settings. They offer researchers, policymakers, and CPs a solid framework to understand specific implementation challenges and develop tailored context-sensitive strategies for sustainable use of the Childcheck.

The identified subgroups are based on the co-occurrence of determinants, aligning with patterns found in prior qualitative research. For example, in the 'CP-client interaction issues' subgroup, we observed the co-occurrence of communication skills, client relationships, and client cooperation. In prior studies, CPs expressed concerns about potential aggressive reactions or damaging their client relationships, leading to reducing parental cooperation, when addressing suspected child abuse. However, such concerns might be caused by lack of communication skills [17, 18]. Similarly, the determinants characterizing the 'RCCAN collaboration issues' subgroup have been previously recognized as co-occurrent [16, 18]. CPs expressed a lack of confidence in follow-up care, with the care offered perceived as inadequate or too slow, potentially exacerbating the child's situation after reporting. These concerns might be influenced by CPs facing challenges related to RCCAN, including a lack of feedback and unclear communication, and often find themselves not taken seriously.

Our findings are comparable to those of Piper et al. (2021), who used LPA to identify subgroups based on pre-implementation determinants [34]. However, differences in innovation type (HIV pre-exposure prophylaxis vs. Childcheck) and implementation phase (pre-implementation vs. active implementation) warrant careful interpretation. Furthermore, Piper et al. focused on organizational readiness for implementation from the perspective of professionals or administrators, while our study centered on the professionals themselves. Piper et al. identified six distinct profiles, including a 'Highest Capacity for Implementation' subgroup, comparable to our ‘Limited implementation issues’ subgroup—both showing high mean scores on implementation determinants. Their 'Resource-Strained Group' aligns with our 'RCCAN collaboration and organizational issues' subgroup, as both faced internal and external organizational barriers and showed the lowest scores on implementation outcomes. Both studies identified a subgroup with neither strongly positive nor negative ratings. Notably, Piper and colleagues were unable to identify a subgroup comparable to our 'CP-client interaction issues' subgroup due to the pre-implementation nature of their study.

While LPA is inherently sample-specific and the exact composition of subgroups may vary across settings, the profiles identified in this study reflect meaningful combinations of implementation determinants—such as knowledge, skills, organizational support, and interorganizational collaboration—that were previously identified in a recent systematic review on domestic violence and child abuse guidelines [35]. This convergence indicates that the challenges captured in our profiles are not isolated or incidental, but part of broader, recurring issues in the field. By identifying subgroups of CPs, this study provides a more nuanced understanding of how implementation determinants tend to co-occur in practice—offering a basis for more tailored implementation strategies.

### Practical implications and future research

By identifying distinct patterns of implementation determinants among CPs—patterns that align with key determinants previously reported in the literature [35]—our study provides a solid foundation for formulating overarching tailored strategies that address multiple, co-occurring barriers in a more integrated and targeted way. It enables organizations to move beyond isolated interventions by aligning their implementation strategies with the specific sets of challenges that tend to co-occur within each subgroup of CPs. For example, in the ‘CP-client interaction issues’ subgroup, CPs might benefit from improving client communication and local consensus discussions to reflect on why the Childcheck is important, rather than a distraction from their “real work”. Meanwhile, the ‘RCCAN collaboration issues’ subgroup could benefit by building partnerships to facilitate information sharing, collaborative problem-solving, and the development of a shared vision and goals related to the implementation of the Childcheck [36]. While these profiles can be broadly informative and applicable across similar settings, organizations seeking to maximize the relevance and effectiveness of their strategies may benefit from conducting a local profile analysis to determine which subgroups are most prominent in their own context. This approach is supported by evidence from a Cochrane Review, which found that tailored strategies improved CPs’ implementation into practice [37]. Successful implementation of guidelines like Childcheck leads to early identification and intervention for children at risk. This, in turn, not only enhances their well-being and reduces the risk of long-term problems [20, 38–40], but also plays a role in constraining associated societal costs [39, 41].

Additionally, our Chi-square analysis revealed that certain subgroups are overrepresented in specific organizations, suggesting that organizational context plays a role in shaping implementation experiences. Organizations should consider these subgroup composition when designing their implementation strategies. For example, forensic MHC settings may need to focus more on improving CP-client interactions, whereas Probation Services might benefit from strengthening collaboration with RCCAN. Although our study captured these patterns of subgroup membership, it does not explicitly model the clustering effects of these subgroups within organizations. Future research could address this limitation by applying multilevel approaches, which would allow for a better understanding of how organizational context influences subgroup distribution and implementation outcomes. The ‘Indifferent attitudes towards implementation’ subgroup primarily comprised CPs working in aMHC settings and demonstrated a low to average implementation level. The term ‘Indifferent attitudes towards implementation’ implies a degree of disinterest among these CPs when confronted with new initiatives or changes. This indifference may be linked to longstanding systemic challenges in Dutch aMHC. The 2015 decentralization led to fragmented care, budget constraints, and complex governance structures, increasing bureaucracy and reducing capacity for new initiatives [32]. Additionally, a 2017 action program aimed at reducing waiting lists, by mid-2022 the list had grown to 80,000 individuals—if which 52% exceeding the 14-week target. [42]. In parallel, the persistent personnel shortage reached 7% of vacant positions in 2022, with an expected continued rise over the next decade [43]. These challenges might have collectively contributed to the observed indifferent attitudes towards Childcheck implementation among CPs in aMHC settings. For a deeper understanding of CPs' indifferent attitudes towards implementing the Childcheck within aMHC, additional qualitative research is essential. Qualitative methods facilitate an in-depth exploration of attitudes, behaviors, and experiences, allowing researchers to delve into the specific contexts and situations influencing indifferent attitudes towards Childcheck implementation.

Furthermore, the data collection period, which spanned from 2019 to 2022, overlapped with the Covid-19 pandemic. This likely disrupted work routines, reduced face-to-face contact, and shifted professional priorities. These changes may have had an impact on the implementation of the Childcheck guideline, and future research should explore how such factors influence guideline implementation and whether the effects observed during this study are specific to the pandemic or represent longer-term trends.

Finally, we want to emphasize that while we clearly observed distinct clusters of determinants in our data—each with their own unique patterns—the exact nature of the interconnection between these determinants and how they collectively influence implementation outcomes remains to be further explored. Our findings provide an important first step in identifying these subgroups, but future research is needed to unravel the precise mechanisms and interactions underlying these patterns.

### Strengths and limitations

This study has several strengths. To the best of our knowledge, it is among the first to take a holistic approach to implementation research, focusing on determinants that CPs perceived as influencing guideline implementation. LPA offers a detailed understanding of how distinct groups of CPs view and experience the determinants affecting the implementation of the Childcheck, offering meaningful insights for practice. Additionally, the study’s sample size of 562 participants exceeds the recommended minimum for LPA, enhancing the robustness of subgroup classification [44]. Moreover, the high entropy of the five-profile model (0.91) indicates well-defined and easily distinguishable profiles, enhancing the validity and interpretability of the subgroup classifications. Last, the use of the MIDI in combination with input from project members and the advisory group provides a well-established, theoretical- and practice-based framework for evaluating implementation determinants.

Limitations should be noted as well. First, the study's reliance on organizational representatives for the distribution of questionnaires introduces a potential limitation in terms of generalizability. The effectiveness of the questionnaire distribution was contingent upon the varying levels of effort and diligence exhibited by these representatives. Additionally, and potentially as a consequence of the preceding limitation, in aMHC, two organizations accounted for ~ 80% of the CP responses. Since the 'Indifferent attitudes towards implementation' subgroup was mainly composed of aMHC CPs, we examined whether this was driven by these two organizations. Analysis showed that CPs from multiple aMHC organizations were present within this subgroup, suggesting a widespread pattern rather than one tied to overrepresented organizations. Still, future studies with more evenly distributed response rates across organizations are needed to confirm the robustness and generalizability of the identified profiles. A second limitation was the lack of data on CPs’ background characteristics, making it impossible to investigate whether factors such as gender, age, or work experience influenced the allocation of CPs in the different subgroups. Similarly, we did not account for fixed organizational characteristics, such as location and size. While our study focused on CPs' perceptions of implementation determinants, it is possible that these fixed organizational factors could have influenced the interconnection between determinants [45, 46]. As we did not include these factors in our analysis, we cannot draw definitive conclusions about their impact. Future research could explore how these organizational characteristics affect the interconnection between implementation determinants and, consequently, implementation outcomes. Third, the study relied on self-report data and utilized a questionnaire with reverse-worded items, potentially introducing response bias that may have affected the identification of latent profiles in LPA. Fourth, while the types of profiles identified in this study reflect determinants previously identified in a systematic review on the implementation of child abuse and domestic violence guidelines [35], the findings are based on data from aMHC and FC settings. It remains unclear whether similar subgroup dynamics exist in other contexts where the Childcheck is implemented, such as emergency departments, ambulance services, or general practices. Moreover, it is uncertain to what extent the identified profiles are specific to the Childcheck intervention, or whether they reflect more general patterns relevant to the implementation of child abuse and domestic violence guidelines. Future research could explore the transferability of these profiles across settings and interventions. Last, we were unable to obtain informed consent, since the implementation impulses were not originally established with a research intention but rather to evaluate, assist, and monitor the implementation of Childcheck. Nevertheless, the study ensured the anonymity of CPs and adhered to ethical guidelines to protect their privacy.

## Conclusion

LPA is a valuable method to capture the heterogeneity in implementation determinants among CPs in aMHC and FC settings. We identified five distinct subgroups, each characterized by its unique set of implementation determinants. Interaction processes between CPs and clients posed significant challenges for a majority of CPs when implementing the Childcheck in practice and should be considered when developing a tailored implementation program. Additionally, considering the low implementation level, CPs facing challenges related to the RCCAN, organizational resources, leadership, and support should not be overlooked, despite being the smallest subgroup. Qualitative research is needed to gain a deeper understanding of the indifferent attitudes towards implementing the Childcheck among CPs in aMHC settings. Recognizing and addressing the specific needs of different CP subgroups, organizations can take more effective steps towards achieving successful guideline implementation and, ultimately, improving the lives of vulnerable children and families.

## Supplementary Information

Supplementary Material 1.

Supplementary Material 2.

Supplementary Material 3.

Supplementary Material 4.

Supplementary Material 5.

Supplementary Material 6.

## Data Availability

All data supporting the conclusions of this study are included in the paper and its additional files. The datasets used and/or analysed during the current study are available from the corresponding author on reasonable request.

## Abbreviations

aMHC: Adult Mental Health Care
CFIR: Consolidated Framework for Implementation Research
CP: Care Professional
FC: Forensic Care
IRT: Item Respons Theory
LPA: Latent Profile Analysis
MIDI: Measurement Instrument for Determinants of Implementation
RCCAN: Reporting Center for Child Abuse and Neglect
