# Supplementary material for: One size fits all? A latent profile analysis to identify care professional subgroups based on implementation determinants
Source: Implement Sci Commun. 2025 Nov 17;6:121. doi: 10.1186/s43058-025-00794-x (PMC12625321; doi:10.1186/s43058-025-00794-x)
Supplement: Supplementary file 2 — Supplementary Material 2. [file 43058_2025_794_MOESM2_ESM.docx]

| **Table B1.** Overview of participating organizations. | | |
| --- | --- | --- |
| **Mental health care** | **Frequency** | **Percent** |
| Altrecht | 4 | 2.1 |
| Dimence | 1 | 0.5 |
| GGZ Friesland | 58 | 29.9 |
| Inforsa Utrecht ambulant | 4 | 2.1 |
| Lentis | 1 | 0.5 |
| Parnassia Groep | 1 | 0.5 |
| Pro Persona | 100 | 51.5 |
| Sanitas Kliniek afdeling psychiatrie | 8 | 4.1 |
| Stichting Bartimeus Sonneheerdt | 1 | 0.5 |
| Stichting de Hezenberg | 4 | 2.1 |
| Trubendorffer | 11 | 5.7 |
| Vincent van Gogh voor Geestelijke Gezondheidszorg | 1 | 0.5 |
| **Forensic mental health care** | **Frequency** | **Percent** |
| De Waag Amersfoort | 2 | 1.1 |
| De Waag Den Haag | 21 | 11.7 |
| De Waag Groningen | 2 | 1.1 |
| De Waag Haarlem | 1 | 0.6 |
| De Waag Zaandam | 8 | 4.4 |
| Fivoor | 41 | 22.8 |
| Fivoor Domein ambulant. AC Leiden en AC Gouda | 9 | 5 |
| Fivoor Domein OFZ klinisch Den Haag | 12 | 6.7 |
| Fivoor Domein TBS (locatie Poortugaal= FPC de Kijvelanden) | 6 | 3.3 |
| Forensisch Centrum locatie De Strandwal - Heiloo | 11 | 6.1 |
| Forensische Psychiatrische Polikliniek. Inforsa | 10 | 5.6 |
| FPA (forensisch psychiatrische afdeling) Transfore Almelo | 5 | 2.8 |
| FPC Oostvaarderskliniek | 1 | 0.6 |
| FPC Oostvaarderskliniek Verslavingsafdeling Basalt | 1 | 0.6 |
| Pompestichting / ForFACT Arnhem/ Nijmegen | 5 | 2.8 |
| Pompestichting / Kairos ForFACT Tiel | 7 | 3.9 |
| Pompestichting / Kairos Nijmegen | 29 | 16.1 |
| TBS de Rooyse wissel | 5 | 2.8 |
| Van der Hoeven Kliniek Utrecht de Waag | 4 | 2.2 |
| **Probation Service** | **Frequency** | **Percent** |
| Intramuraal Motivatie Centrum (IMC) | 10 | 6.3 |
| Reclassering Inforsa | 10 | 6.3 |
| Reclassering Inforsa Amsterdam | 13 | 8.2 |
| Reclassering Regio Noord-West | 42 | 26.4 |
| Reclassering Regio Zuid-West | 23 | 14.5 |
| Vincent van Gogh reclassering | 1 | 0.6 |
| VNN forensische polikliniek | 8 | 5 |
| VNN reclassering Drenthe | 15 | 9.4 |
| VNN reclassering Friesland | 18 | 11.3 |
| VNN reclassering Groningen | 19 | 11.9 |
| **The Salvation Army** | **Frequency** | **Percent** |
| Leger des Heils Regio Rotterdam | 13 | 43.3 |
| Leger des Heils W&G Zuidoost | 1 | 3.3 |
| Leger des Heils Jeugd Midden Nederland | 16 | 53.3 |
