# Supplementary material for: One size fits all? A latent profile analysis to identify care professional subgroups based on implementation determinants
Source: Implement Sci Commun. 2025 Nov 17;6:121. doi: 10.1186/s43058-025-00794-x (PMC12625321; doi:10.1186/s43058-025-00794-x)
Supplement: Supplementary file 3 — Supplementary Material 3. [file 43058_2025_794_MOESM3_ESM.docx]

| **Table C1.** Items included in the questionnaire to identify implementation determinants and assess implementation level of care professionals. | | | | |
| --- | --- | --- | --- | --- |
| **Determinant** | | **Variable** | **Measurement scale (5-point)** | **Source** |
| **Innovation** | Compatibility | Inquiring about the ChildCheck aligns well with my client conversation | Strongly disagree – Strongly agree | MIDI |
|  | Observability | My clients seem satisfied with the assistance offered by RCCAN | Strongly disagree – Strongly agree | MIDI |
|  | Procedural clarity | I have a clear understanding of what the ChildCheck entails | Strongly disagree – Strongly agree | MIDI |
|  | Relative priority | Other tasks I need to perform obstruct the inquiry of the ChildCheck * | Strongly disagree – Strongly agree | SDI |
| **Organization** | Coordinator | The contact person is easily accessible to me for advice | Strongly disagree – Strongly agree | MIDI |
|  | Coordinator | I have had positive experiences in seeking advice from the contact person | Strongly disagree – Strongly agree | MIDI |
|  | Financial resources | There are sufficient financial resources available within my organization to carry out the ChildCheck effectively | Strongly disagree – Strongly agree | MIDI |
|  | Formal ratification | In my organization, there are formal agreements regarding the implementation of the ChildCheck | Strongly disagree – Strongly agree | MIDI |
|  | Partnership and connections | RCCAN is a good partner for discussions from my perspective | Strongly disagree – Strongly agree | SDI |
|  | Partnership and connections | RCCAN is easily reachable for advice in my opinion | Strongly disagree – Strongly agree | SDI |
|  | Partnership and connections | I am satisfied with the collaboration with RCCAN concerning coordinating appropriate assistance for my clients | Strongly disagree – Strongly agree | SDI |
|  | Partnership and connections | I am content with the feedback I receive from RCCAN after the completion of investigations following reports | Strongly disagree – Strongly agree | SDI |
|  | Time | I have sufficient time to conduct the ChildCheck effectively | Strongly disagree – Strongly agree | MIDI |
| **Professional** | Access to knowledge | In my organization, I receive adequate training to perform the ChildCheck effectively | Strongly disagree – Strongly agree | SDI |
|  | Client cooperation ChildCheck | Clients often react negatively to the inquiry of the ChildCheck* | Strongly disagree – Strongly agree | MIDI |
|  | Client cooperation RCCAN | Clients generally understand when I explain that I will make a report to RCCAN | Strongly disagree – Strongly agree | MIDI |
|  | Client cooperation RCCAN | Clients generally react negatively when I explain that I will make a report to RCCAN* | Strongly disagree – Strongly agree | MIDI |
|  | Communication skills | I find it challenging to bring up the ChildCheck | Strongly disagree – Strongly agree | SDI |
|  | Descriptive norm | Is the ChildCheck applied within your organization? | Strongly disagree – Strongly agree | MIDI |
|  | Descriptive norm | Are there colleagues within your team who apply the ChildCheck? | Strongly disagree – Strongly agree | MIDI |
|  | General skills | I possess the necessary skills to conduct the ChildCheck effectively | Strongly disagree – Strongly agree | SDI |
|  | Implementation needs | I require (more) support to conduct the ChildCheck effectively* | Strongly disagree – Strongly agree | SDI |
|  | Knowledge | I know how to inquire about the ChildCheck | Strongly disagree – Strongly agree | MIDI |
|  | Knowledge | I know how and where to record the results of the ChildCheck | Strongly disagree – Strongly agree | MIDI |
|  | Professional obligation | I understand the importance of conducting the ChildCheck | Strongly disagree – Strongly agree | MIDI |
|  | Professional obligation | I am motivated to conduct the ChildCheck effectively | Strongly disagree – Strongly agree | MIDI |
|  | Professional obligation | I consider it my duty to perform a ChildCheck at every intake with a new client | Strongly disagree – Strongly agree | MIDI |
|  | Professional obligation | I conduct the ChildCheck because I feel responsible for the well-being and safety of my client's children | Strongly disagree – Strongly agree | MIDI |
|  | Relationship client | I fear damaging my client relationship if I apply the ChildCheck* | Strongly disagree – Strongly agree | SDI |
|  | Routine | Conducting the ChildCheck has become a habit for me; it's something I have made my own | Strongly disagree – Strongly agree | SDI |
|  | Outcome expectations | I have confidence that my clients will receive appropriate assistance from RCCAN | Strongly disagree – Strongly agree | MIDI |
|  | Outcome expectations | I have confidence that my clients will receive TIMELY appropriate assistance from RCCAN | Strongly disagree – Strongly agree | MIDI |
|  | Social support | I perceive the collaboration with colleagues/supervisors regarding the implementation of the ChildCheck as positive | Strongly disagree – Strongly agree | MIDI |
|  | Implementation level | Do you personally apply the ChildCheck? | Never – Always (4-point) | SDI |
|  | Implementation level | Do you apply the ChildCheck in the initial client meeting? | Never – Always (4-point) | SDI |
|  | Implementation level | Do you apply the ChildCheck in every evaluation of treatment/risk assessment and/or interim concerns with clients? | Never – Always (4-point) | SDI |
|  | Implementation level† | I conduct the ChildCheck in accordance with step 1 of the Reporting Code | Strongly disagree – Strongly agree | SDI |
| *Note*: Items marked with an asterisk (*) indicate negative-worded items; MIDI=Measurement of Implementation Determinants of Innovations; SDI=Self-developed item derived from project group evaluations; † item not included in the construct. | | | | |
