# Supplementary material for: One size fits all? A latent profile analysis to identify care professional subgroups based on implementation determinants
Source: Implement Sci Commun. 2025 Nov 17;6:121. doi: 10.1186/s43058-025-00794-x (PMC12625321; doi:10.1186/s43058-025-00794-x)
Supplement: Supplementary file 4 — Supplementary Material 4. [file 43058_2025_794_MOESM4_ESM.docx]

## Creating subsets for the different constructs

```{r}

SS.ClientCoop <- subset(ZET_Data_Merged, select = c('R_V44', 'RN_V45'))

SS.DescrNorm <- subset(ZET_Data_Merged, select = c('V24', 'V25'))

SS.Knowledge <- subset(ZET_Data_Merged, select = c('R_V31', 'R_V32'))

SS.OutcomeExp <- subset(ZET_Data_Merged, select = c('R_V49', 'R_V50'))

SS.ProfObl <- subset(ZET_Data_Merged, select = c('R_V35', 'R_V36', 'R_V38', 'R_V39'))

SS.Coordinator <- subset(ZET_Data_Merged, select = c('R_V42', 'R_V43'))

SS.Partnership <- subset(ZET_Data_Merged, select = c('R_V46', 'R_V47', 'R_V48', 'R_V53'))

SS.ImpLevel <- subset(ZET_Data_Merged, select = c('V26', 'V27', 'V28', 'V30'))

```

## Item Response Theory for the different constructs

```{r}

# irt analysis with client cooperation

Dimension.ClientCoop = mirt::mirt(SS.ClientCoop, model = 1, itemtype = "gpcm")

PersonScores.Scale.ClientCoop = fscores(Dimension.ClientCoop)

summary(PersonScores.Scale.ClientCoop)

summary(Dimension.ClientCoop)

coef(Dimension.ClientCoop, IRTpars = TRUE)

itemplot(Dimension.ClientCoop, 1, type = "trace")

itemplot(Dimension.ClientCoop, 1, type = "info")

plot(Dimension.ClientCoop, type = "trace")

plot(Dimension.ClientCoop, type = "info")

# irt analysis with descriptive norm

Dimension.DescrNorm = mirt::mirt(SS.DescrNorm, model = 1, itemtype = "gpcm")

PersonScores.Scale.DescrNorm = fscores(Dimension.DescrNorm)

summary(PersonScores.Scale.DescrNorm)

summary(Dimension.DescrNorm)

coef(Dimension.DescrNorm, IRTpars = TRUE)

itemplot(Dimension.DescrNorm, 1, type = "trace")

itemplot(Dimension.DescrNorm, 1, type = "info")

plot(Dimension.DescrNorm, type = "trace")

plot(Dimension.DescrNorm, type = "info")

# irt analysis with knowledge

Dimension.Knowledge = mirt::mirt(SS.Knowledge, model = 1, itemtype = "gpcm")

PersonScores.Scale.Knowledge = fscores(Dimension.Knowledge)

summary(PersonScores.Scale.Knowledge)

summary(Dimension.Knowledge)

coef(Dimension.Knowledge, IRTpars = TRUE)

itemplot(Dimension.Knowledge, 1, type = "trace")

itemplot(Dimension.Knowledge, 1, type = "info")

plot(Dimension.Knowledge, type = "trace")

plot(Dimension.Knowledge, type = "info")

# irt analysis with outcome expectations

Dimension.OutcomeExp = mirt::mirt(SS.OutcomeExp, model = 1, itemtype = "gpcm")

PersonScores.Scale.OutcomeExp = fscores(Dimension.OutcomeExp)

summary(PersonScores.Scale.OutcomeExp)

summary(Dimension.OutcomeExp)

coef(Dimension.OutcomeExp, IRTpars = TRUE)

itemplot(Dimension.OutcomeExp, 1, type = "trace")

itemplot(Dimension.OutcomeExp, 1, type = "info")

plot(Dimension.OutcomeExp, type = "trace")

plot(Dimension.OutcomeExp, type = "info")

# irt analysis with professional obligation

Dimension.ProfObl = mirt::mirt(SS.ProfObl, model = 1, itemtype = "gpcm")

PersonScores.Scale.ProfObl = fscores(Dimension.ProfObl)

summary(PersonScores.Scale.ProfObl)

summary(Dimension.ProfObl)

coef(Dimension.ProfObl, IRTpars = TRUE)

itemplot(Dimension.ProfObl, 1, type = "trace")

itemplot(Dimension.ProfObl, 1, type = "info")

plot(Dimension.ProfObl, type = "trace")

plot(Dimension.ProfObl, type = "info")

# irt analysis with coordinator

Dimension.Coordinator = mirt::mirt(SS.Coordinator, model = 1, itemtype = "gpcm")

PersonScores.Scale.Coordinator = fscores(Dimension.Coordinator)

summary(PersonScores.Scale.Coordinator)

summary(Dimension.Coordinator)

coef(Dimension.Coordinator, IRTpars = TRUE)

itemplot(Dimension.Coordinator, 1, type = "trace")

itemplot(Dimension.Coordinator, 1, type = "info")

plot(Dimension.Coordinator, type = "trace")

plot(Dimension.Coordinator, type = "info")

# irt analysis with partnership & Connections

Dimension.Partnership = mirt::mirt(SS.Partnership, model = 1, itemtype = "gpcm")

PersonScores.Scale.Partnership = fscores(Dimension.Partnership)

summary(PersonScores.Scale.Partnership)

summary(Dimension.Partnership)

coef(Dimension.Partnership, IRTpars = TRUE)

itemplot(Dimension.Partnership, 1, type = "trace")

itemplot(Dimension.Partnership, 1, type = "info")

plot(Dimension.Partnership, type = "trace")

plot(Dimension.Partnership, type = "info")

# irt analysis with implementation level

Dimension.ImpLevel = mirt::mirt(SS.ImpLevel, model = 1, itemtype = "gpcm")

PersonScores.Scale.ImpLevel = fscores(Dimension.ImpLevel)

summary(Dimension.ImpLevel)

coef(Dimension.ImpLevel, IRTpars = TRUE)

itemplot(Dimension.ImpLevel, 1, type = "trace")

itemplot(Dimension.ImpLevel, 1, type = "info")

plot(Dimension.ImpLevel, type = "trace")

plot(Dimension.ImpLevel, type = "info")

```

## Based on the above, create new subsets for the different constructs

```{r}

SS.ClientCoop <- subset(ZET_Data_Merged, select = c('R_V44', 'RN_V45'))

SS.DescrNorm <- subset(ZET_Data_Merged, select = c('V24', 'V25'))

SS.Knowledge <- subset(ZET_Data_Merged, select = c('R_V31', 'R_V32'))

SS.OutcomeExp <- subset(ZET_Data_Merged, select = c('R_V49', 'R_V50'))

SS.ProfObl <- subset(ZET_Data_Merged, select = c('R_V35', 'R_V36', 'R_V38', 'R_V39'))

SS.Coordinator <- subset(ZET_Data_Merged, select = c('R_V42', 'R_V43'))

SS.Partnership <- subset(ZET_Data_Merged, select = c('R_V46', 'R_V47', 'R_V48', 'R_V53'))

SS.ImpLevel <- subset(ZET_Data_Merged, select = c('V26', 'V27', 'V28'))

# V38 V39 V42 V53 were recoded to 3 categories based on IRT.

# V30 was removed from the construct implementation level.

```

## Item Response Theory for the new constructs

```{r}

# irt analysis with client cooperation

Dimension.ClientCoop = mirt::mirt(SS.ClientCoop, model = 1, itemtype = "gpcm")

PersonScores.Scale.ClientCoop = fscores(Dimension.ClientCoop)

summary(PersonScores.Scale.ClientCoop)

summary(Dimension.ClientCoop)

coef(Dimension.ClientCoop, IRTpars = TRUE)

itemplot(Dimension.ClientCoop, 1, type = "trace")

itemplot(Dimension.ClientCoop, 1, type = "info")

plot(Dimension.ClientCoop, type = "trace")

plot(Dimension.ClientCoop, type = "info")

# irt analysis with descriptive norm

Dimension.DescrNorm = mirt::mirt(SS.DescrNorm, model = 1, itemtype = "gpcm")

PersonScores.Scale.DescrNorm = fscores(Dimension.DescrNorm)

summary(PersonScores.Scale.DescrNorm)

summary(Dimension.DescrNorm)

coef(Dimension.DescrNorm, IRTpars = TRUE)

itemplot(Dimension.DescrNorm, 1, type = "trace")

itemplot(Dimension.DescrNorm, 1, type = "info")

plot(Dimension.DescrNorm, type = "trace")

plot(Dimension.DescrNorm, type = "info")

# irt analysis with knowledge

Dimension.Knowledge = mirt::mirt(SS.Knowledge, model = 1, itemtype = "gpcm")

PersonScores.Scale.Knowledge = fscores(Dimension.Knowledge)

summary(PersonScores.Scale.Knowledge)

summary(Dimension.Knowledge)

coef(Dimension.Knowledge, IRTpars = TRUE)

itemplot(Dimension.Knowledge, 1, type = "trace")

itemplot(Dimension.Knowledge, 1, type = "info")

plot(Dimension.Knowledge, type = "trace")

plot(Dimension.Knowledge, type = "info")

# irt analysis with outcome expectations

Dimension.OutcomeExp = mirt::mirt(SS.OutcomeExp, model = 1, itemtype = "gpcm")

PersonScores.Scale.OutcomeExp = fscores(Dimension.OutcomeExp)

summary(PersonScores.Scale.OutcomeExp)

summary(Dimension.OutcomeExp)

coef(Dimension.OutcomeExp, IRTpars = TRUE)

itemplot(Dimension.OutcomeExp, 1, type = "trace")

itemplot(Dimension.OutcomeExp, 1, type = "info")

plot(Dimension.OutcomeExp, type = "trace")

plot(Dimension.OutcomeExp, type = "info")

# irt analysis with professional obligation

Dimension.ProfObl = mirt::mirt(SS.ProfObl, model = 1, itemtype = "gpcm")

PersonScores.Scale.ProfObl = fscores(Dimension.ProfObl)

summary(PersonScores.Scale.ProfObl)

summary(Dimension.ProfObl)

coef(Dimension.ProfObl, IRTpars = TRUE)

itemplot(Dimension.ProfObl, 1, type = "trace")

itemplot(Dimension.ProfObl, 1, type = "info")

plot(Dimension.ProfObl, type = "trace")

plot(Dimension.ProfObl, type = "info")

# irt analysis with coordinator

Dimension.Coordinator = mirt::mirt(SS.Coordinator, model = 1, itemtype = "gpcm")

PersonScores.Scale.Coordinator = fscores(Dimension.Coordinator)

summary(PersonScores.Scale.Coordinator)

summary(Dimension.Coordinator)

coef(Dimension.Coordinator, IRTpars = TRUE)

itemplot(Dimension.Coordinator, 1, type = "trace")

itemplot(Dimension.Coordinator, 1, type = "info")

plot(Dimension.Coordinator, type = "trace")

plot(Dimension.Coordinator, type = "info")

# irt analysis with partnership & Connections

Dimension.Partnership = mirt::mirt(SS.Partnership, model = 1, itemtype = "gpcm")

PersonScores.Scale.Partnership = fscores(Dimension.Partnership)

summary(PersonScores.Scale.Partnership)

summary(Dimension.Partnership)

coef(Dimension.Partnership, IRTpars = TRUE)

itemplot(Dimension.Partnership, 1, type = "trace")

itemplot(Dimension.Partnership, 1, type = "info")

plot(Dimension.Partnership, type = "trace")

plot(Dimension.Partnership, type = "info")

# irt analysis with implementation level

Dimension.ImpLevel = mirt::mirt(SS.ImpLevel, model = 1, itemtype = "gpcm")

PersonScores.Scale.ImpLevel = fscores(Dimension.ImpLevel)

summary(Dimension.ImpLevel)

coef(Dimension.ImpLevel, IRTpars = TRUE)

itemplot(Dimension.ImpLevel, 1, type = "trace")

itemplot(Dimension.ImpLevel, 1, type = "info")

plot(Dimension.ImpLevel, type = "trace")

plot(Dimension.ImpLevel, type = "info")

```

## Create new dataset

```{r}

Dataset.Variabelen.mirt = cbind(PersonScores.Scale.ClientCoop, PersonScores.Scale.Coordinator, PersonScores.Scale.DescrNorm, PersonScores.Scale.ImpLevel, PersonScores.Scale.Knowledge, PersonScores.Scale.OutcomeExp, PersonScores.Scale.Partnership, PersonScores.Scale.ProfObl)

colnames(Dataset.Variabelen.mirt) <- c("ClientCoorperation_RCCAN", "Coordinator", "DescriptiveNorm", "ImplementationLevel", "Knowledge", "OutcomeExpectations", "PartnershipConnections", "ProfessionalObligation")

write.table(Dataset.Variabelen.mirt, file = "Dataset_mirt_vars.csv", sep = ",", row.names = TRUE)

Dataset.LCA.compleet = cbind(ZET_Data_Merged, Dataset.Variabelen.mirt)

Dataset.LCA.compleet$Organisatie <- NULL

Dataset.LCA.compleet$Afdeling <- NULL

Dataset.LCA.compleet$R_V44 <- NULL

Dataset.LCA.compleet$RN_V45 <- NULL

Dataset.LCA.compleet$V24 <- NULL

Dataset.LCA.compleet$V25 <- NULL

Dataset.LCA.compleet$R_V31 <- NULL

Dataset.LCA.compleet$R_V32 <- NULL

Dataset.LCA.compleet$R_V49 <- NULL

Dataset.LCA.compleet$R_V50 <- NULL

Dataset.LCA.compleet$R_V35 <- NULL

Dataset.LCA.compleet$R_V36 <- NULL

Dataset.LCA.compleet$RIRT_V38 <- NULL

Dataset.LCA.compleet$RIRT_V39 <- NULL

Dataset.LCA.compleet$RIRT_V42 <- NULL

Dataset.LCA.compleet$R_V43 <- NULL

Dataset.LCA.compleet$R_V46 <- NULL

Dataset.LCA.compleet$R_V47 <- NULL

Dataset.LCA.compleet$R_V48 <- NULL

Dataset.LCA.compleet$RIRT_V53 <- NULL

Dataset.LCA.compleet$V26 <- NULL

Dataset.LCA.compleet$V27 <- NULL

Dataset.LCA.compleet$V28 <- NULL

Dataset.LCA.compleet$R_V30 <- NULL

colnames(Dataset.LCA.compleet) <- c("ID", "Groep_ID", "Groep_IDcat", "Awareness", "ProceduralClarity", "Compatibility", "Skills_Communication", "Skills_General", "RelationshipClient", "ClientCooperation_Childcheck", "Observability", "RelativePriority",

"BehavioralRegulations", "FinancialResources", "Time", "SocialSupport", "FormalRatification", "AccessToKnowledge", "ImplementationNeeds", "ClientCoorperation_RCCAN", "Coordinator", "DescriptiveNorm", "ImplementationLevel", "Knowledge", "OutcomeExpectations", "PartnershipConnections", "ProfessionalObligation")

write.table(Dataset.LCA.compleet, file ="Dataset_LCA.csv", sep = ",", row.names = TRUE)

```

## Rescale IRT person scores

```{r}

data_zet$ClientCoorperation_RCCAN <- scales::rescale(data_zet$ClientCoorperation_RCCAN, to = c(1, 5))

data_zet$Coordinator <- scales::rescale(data_zet$Coordinator, to = c(1, 5))

data_zet$DescriptiveNorm <- scales::rescale(data_zet$DescriptiveNorm, to = c(1, 5))

data_zet$Knowledge <- scales::rescale(data_zet$Knowledge, to = c(1, 5))

data_zet$OutcomeExpectations <- scales::rescale(data_zet$OutcomeExpectations, to = c(1, 5))

data_zet$PartnershipConnections <- scales::rescale(data_zet$PartnershipConnections, to = c(1, 5))

data_zet$ProfessionalObligation <- scales::rescale(data_zet$ProfessionalObligation, to = c(1, 5))

data_zet$ImplementationLevel <- scales::rescale(data_zet$ImplementationLevel, to = c(1, 5))

```

| **Item parameters** | | | | | |
| --- | --- | --- | --- | --- | --- |
| **Item** | **a** | **b1** | **b2** | **b3** | **b4** |
| *Client cooperation* |  |  |  |  |  |
| Clients generally understand when I explain to them that I am going to make a report to RCCAN | -4.359 | 0.835 | 0.291 | -0.707 | -1.514 |
| Clients generally react negatively when I explain to them that I am going to make a report to RCCAN | 1.050 | -0.841 | -0.765 | 1.296 | 0.095 |
| *Descriptive norm* |  |  |  |  |  |
| Is the Childcheck applied within your organization? | 4.762 | -2.193 | -0.527 | 0.732 |  |
| Are there colleagues within your team who apply the Childcheck? | 3.518 | -2.025 | -0.426 | 1.010 |  |
| *Knowledge* |  |  |  |  |  |
| I know how to conduct the Childcheck | 1.562 | -2.061 | -1.359 | -0.264 | 0.524 |
| I know how and where to document the outcomes of the Childcheck | 4.058 | -1.717 | -1.088 | -0.038 | 0.574 |
| *Outcome expectations* |  |  |  |  |  |
| I am confident that my clients will receive appropriate support from RCCAN | 2.114 | -1.317 | -1.127 | 0.045 | 1.146 |
| I am confident that my clients will receive appropriate support from RCCAN in a timely manner | 7.332 | -1.232 | -0.666 | 0.345 | 1.400 |
| *Professional obligation* |  |  |  |  |  |
| I consider it my responsibility to conduct a Childcheck during every intake with a new client | 1.668 | -1.244 | -3.597 | -0.059 | -0.398 |
| I conduct the Childcheck because I feel responsible for the well-being and safety of my clients’ children | 1.543 | -1.603 | -3.793 | -0.238 | -0.208 |
| I understand the importance of conducting the Childcheck | 1.302 | -2.183 | -0.598 |  |  |
| I am motivated to conduct the Childcheck properly | 1.590 | -2.468 | -0.557 |  |  |
| *Coordinator* |  |  |  |  |  |
| The aandachtsfunctionaris (confidential advisor) is easy for me to contact for advice | 1.381 | -1.841 | 0.303 |  |  |
| I have had good experiences when consulting the aandachtsfunctionaris for advice | 10.045 | -1.009 | -1.064 | 0.369 | 0.93 |
| *Partnership and connections* |  |  |  |  |  |
| I am satisfied with the collaboration with RCCAN when it comes to coordinating appropriate support for my clients | 2.998 | -1.049 | -1.383 | -0.258 | 0.600 |
| I am satisfied with the feedback I receive from RCCAN after reports or investigations | 1.864 | -0.624 | -1.828 | -0.266 | 0.611 |
| I consider RCCAN to be a good partner for consultations | 2.521 | -0.830 | -1.200 | 0.130 | 1.055 |
| RCCAN is easily accessible to me for advice | 1.239 | -0.659 | 0.441 |  |  |
| *Implementation level* |  |  |  |  |  |
| Do you apply the Childcheck yourself? | 2.185 | -1.151 | -0.253 | 0.689 |  |
| Do you apply the Childcheck during the first meeting with clients? | 2.090 | -0.588 | 0.028 | 0.783 |  |
| Do you apply the Childcheck during every evaluation/risk assessment and/or when new concerns arise with clients? | 1.272 | -0.474 | 0.602 | 1.490 |  |


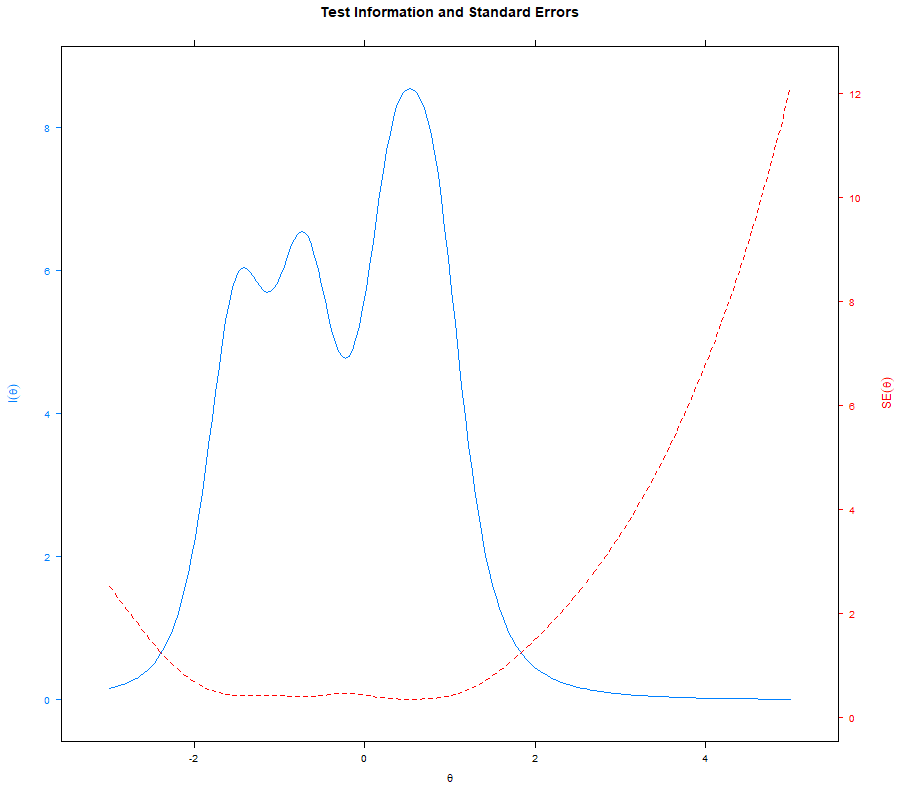


Client cooperation

Descriptive norm


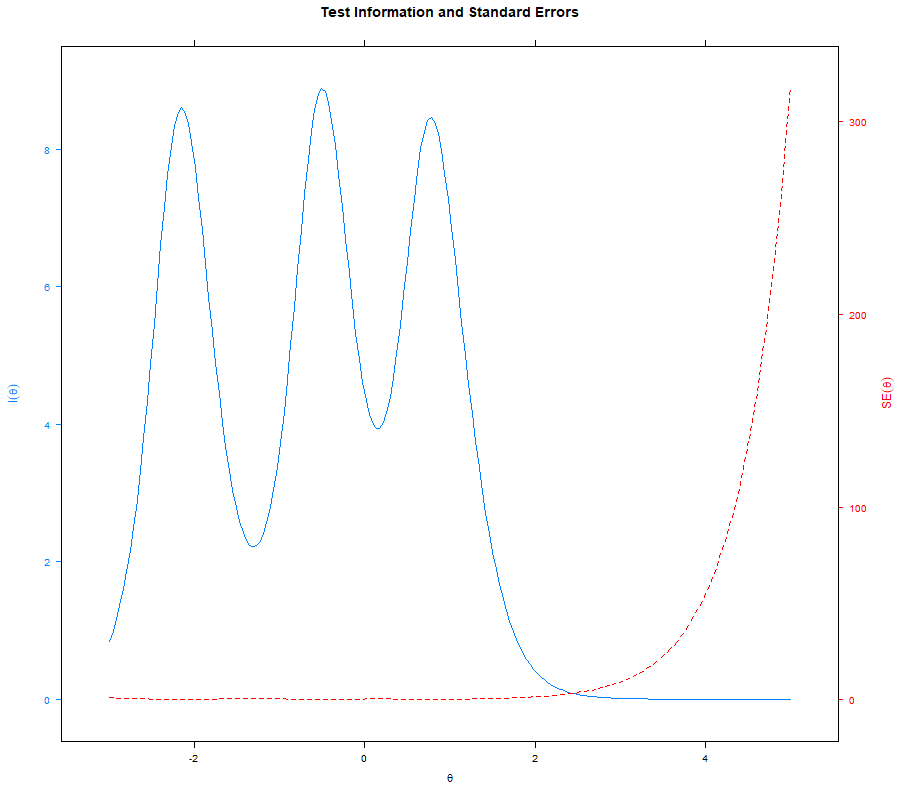


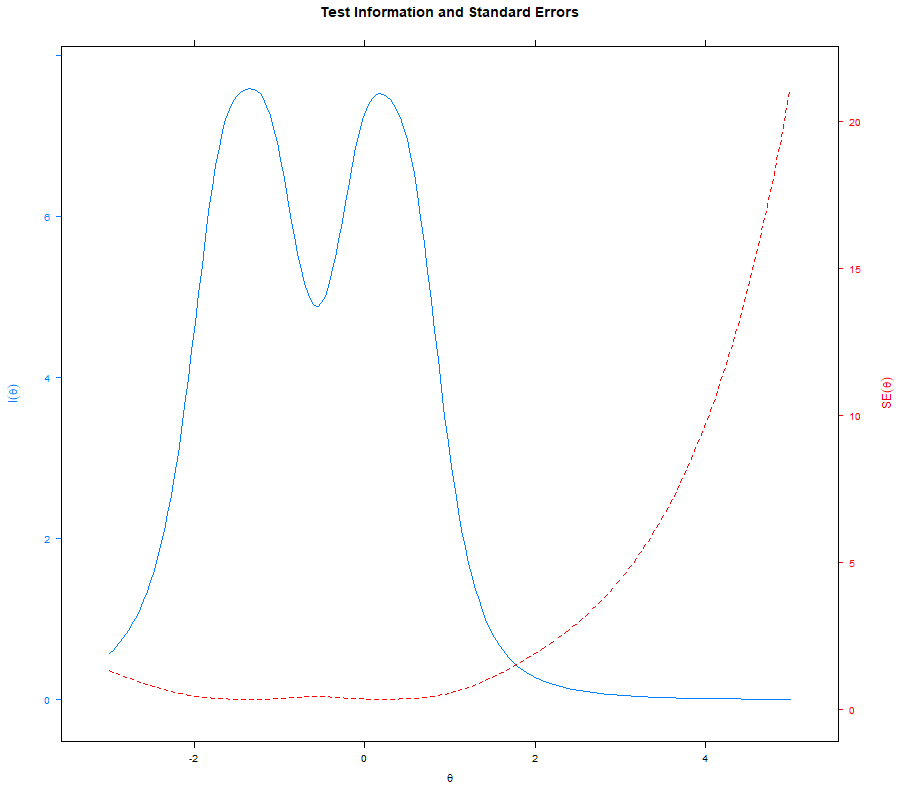

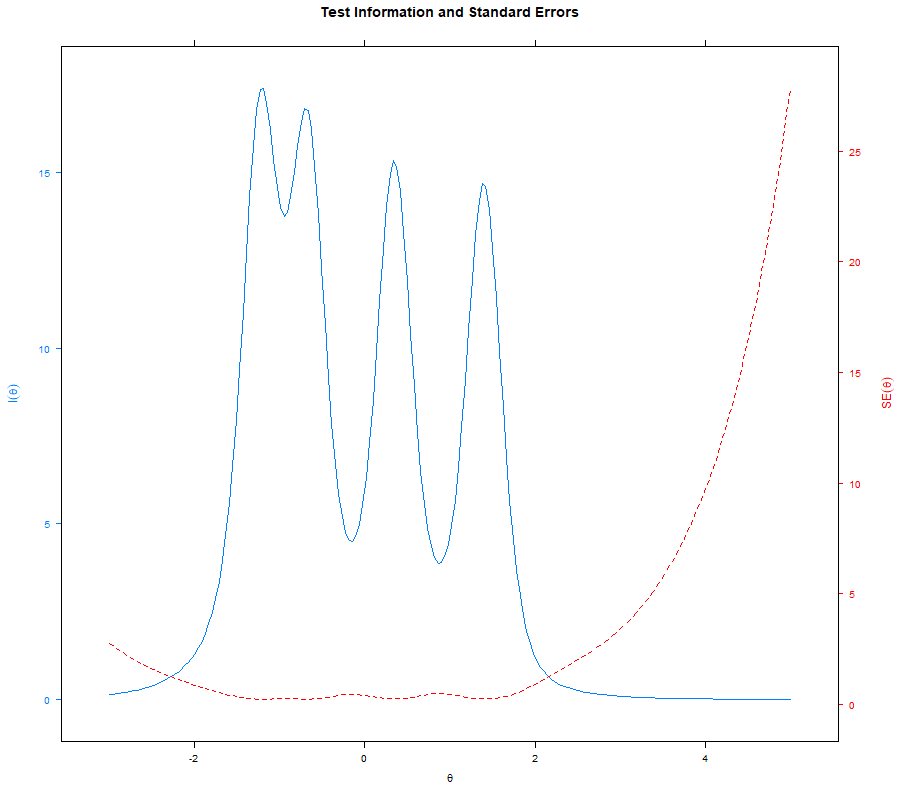


Knowledge

Outcome expectation


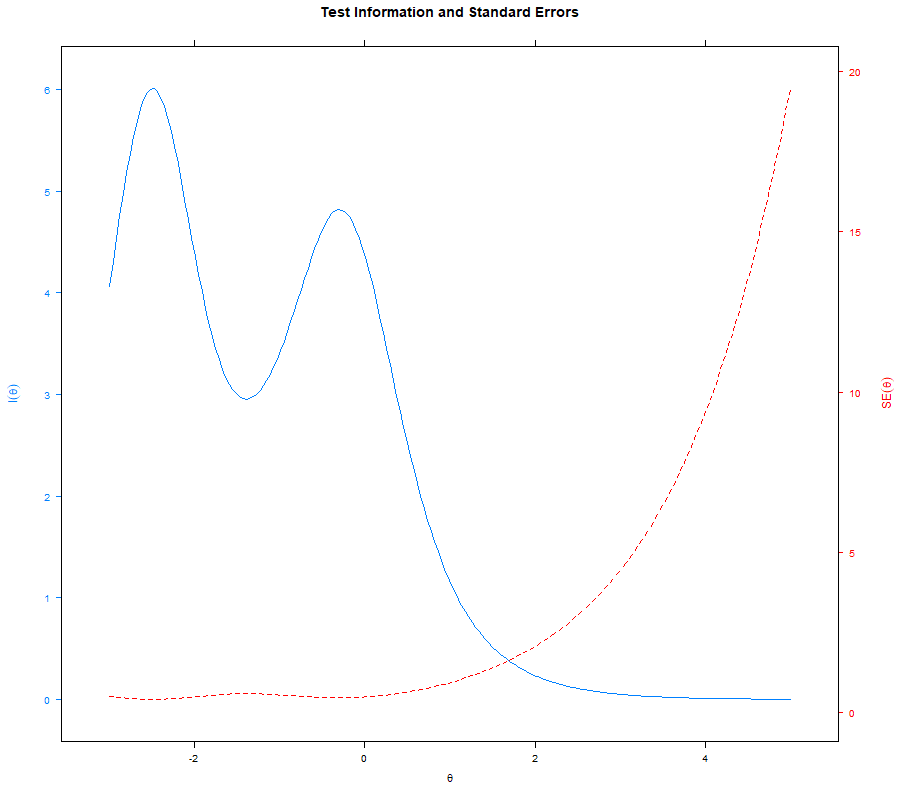

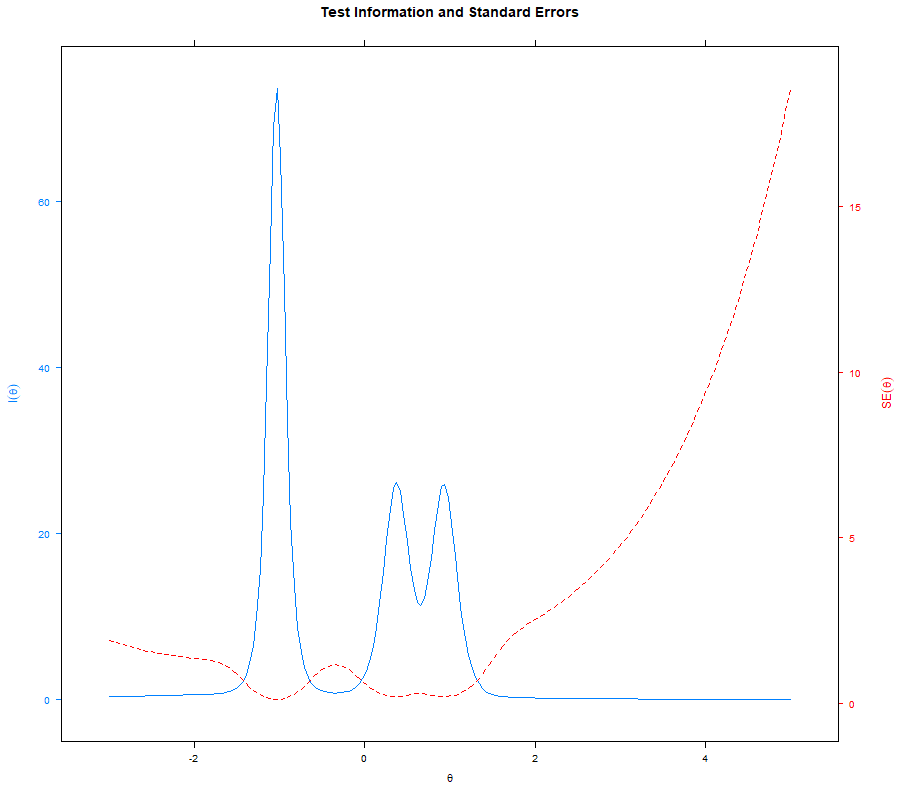

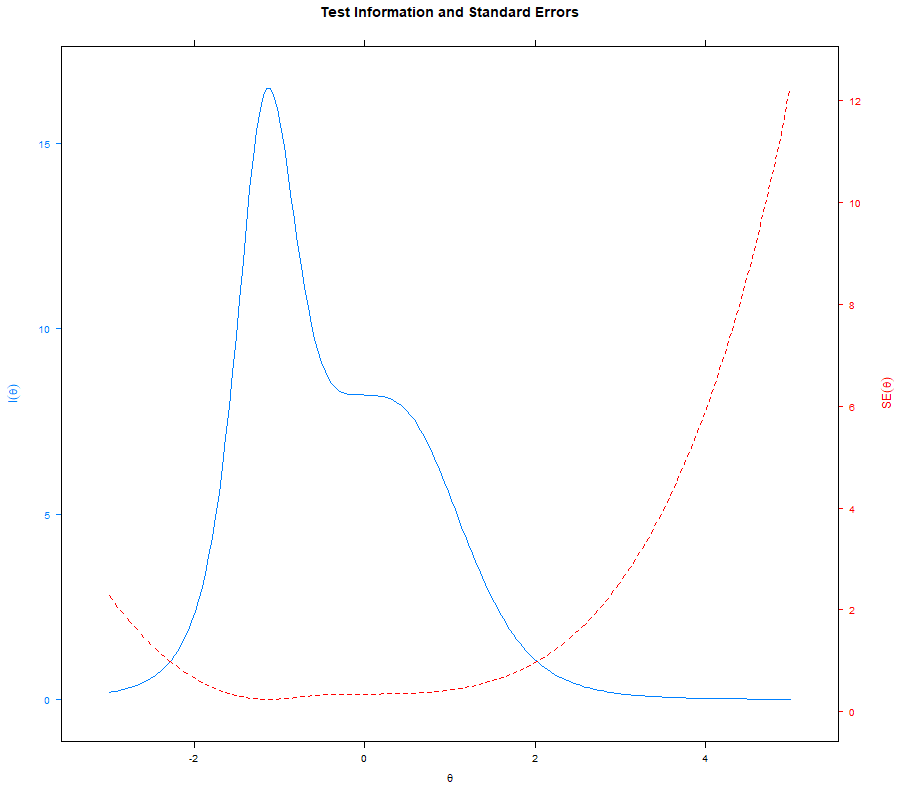

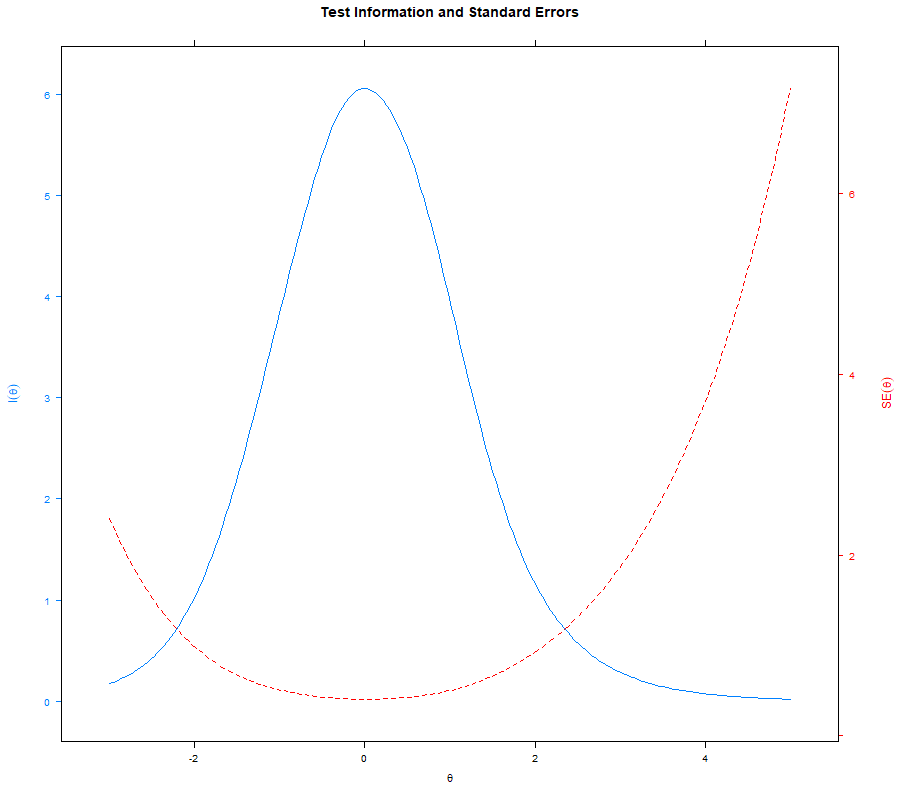


Professional obligation

Coordinator

Partnership and connections

Implementation level
