# Supplementary material for: One size fits all? A latent profile analysis to identify care professional subgroups based on implementation determinants
Source: Implement Sci Commun. 2025 Nov 17;6:121. doi: 10.1186/s43058-025-00794-x (PMC12625321; doi:10.1186/s43058-025-00794-x)
Supplement: Supplementary file 5 — Supplementary Material 5. [file 43058_2025_794_MOESM5_ESM.docx]

##### CFA for implementation determinants #####

```{r CFA determinants}

# CFA model

model <- '

# Construct: Client Cooperation (ClientCoop)

ClientCoop =~ R_V44 + RN_V45_Recoded

# Construct: Descriptive Norm (DescrNorm)

DescrNorm =~ V24 + V25

# Construct: Knowledge

Knowledge =~ R_V31 + R_V32

# Construct: Outcome Expectations (OutcomeExp)

OutcomeExp =~ R_V49 + R_V50

# Construct: Professional Obligation (ProfObl)

ProfObl =~ R_V35 + R_V36 + RIRT_V38 + RIRT_V39

# Construct: Coordinator

Coordinator =~ RIRT_V42 + R_V43

# Construct: Partnership and connections

Partnership =~ R_V46 + R_V47 + R_V48 + RIRT_V53

'

# CFA

Fit_ImpDeter <- cfa(model, data = ZET_Data_Merged)

# Model summary

summary_fit <- summary(fit_ImpDeter, fit.measures = TRUE, standardized = TRUE)

summary_fit

# Fit indices

fit_indices <- summary_fit$fit[c("chisq", "df", "pvalue", "cfi", "tli", "rmsea", "srmr")]

print(fit_indices)

# Extract standaardized solutions

standardized_solution <- standardizedSolution(fit_ImpDeter)

standardized_solution

# calculate R2 values

r_squared <- inspect(fit_ImpDeter, what = "r2")

# Create a data frame for the factor loadings

factor_loadings <- data.frame(

Latente_Variabele = standardized_solution$lhs,

Item = standardized_solution$rhs,

Estimate = standardized_solution$est, # Unstandardized estimates

SE = standardized_solution$se, # Standard errors

Z = standardized_solution$z, # Z-values

Lambda = standardized_solution$est.std # Standardized factor loadings

)

# Filter the appropriate rows for factor loadings

factor_loadings <- factor_loadings[standardized_solution$op == "=~", ]

# Add R² values to the data frame

factor_loadings$R2 <- r_squared[match(factor_loadings$Item, names(r_squared))]

# Add p-values to the data frame

factor_loadings$p_value <- 2 * (1 - pnorm(abs(factor_loadings$Z)))

# Rearranging the columns to desired order

factor_loadings <- factor_loadings[, c("Latente_Variabele", "Item", "Estimate", "SE", "Z", "Lambda", "R2", "p_value")]

# Print the table with factor loadings and R² values

print(factor_loadings)

# Reliability

composite_reliability <- compRelSEM(fit_ImpDeter)

print(composite_reliability)

```

##### CFA for implementation level #####

```{r CFA implementation level}

# CFA model

model <- '

# Construct: Implementation Level (ImpLevel)

ImpLevel =~ V26 + V27 + V28

'

# CFA

Fit_ImpLevel <- cfa(model, data = ZET_Data_Merged)

# Model summary

summary_fit <- summary(fit_ImpLevel, fit.measures = TRUE, standardized = TRUE)

summary_fit

# Fit indices

fit_indices <- summary_fit$fit[c("chisq", "df", "pvalue", "cfi", "tli", "rmsea", "srmr")]

print(fit_indices)

# Extract standaardized solutions

standardized_solution <- standardizedSolution(fit_ImpLevel)

standardized_solution

# calculate R2 values

r_squared <- inspect(fit_ImpLevel, what = "r2")

# Create a data frame for the factor loadings

factor_loadings <- data.frame(

Latente_Variabele = standardized_solution$lhs,

Item = standardized_solution$rhs,

Estimate = standardized_solution$est, # Unstandardized estimates

SE = standardized_solution$se, # Standard errors

Z = standardized_solution$z, # Z-values

Lambda = standardized_solution$est.std # Standardized factor loadings

)

# Filter the appropriate rows for factor loadings

factor_loadings <- factor_loadings[standardized_solution$op == "=~", ]

# Add R² values to the data frame

factor_loadings$R2 <- r_squared[match(factor_loadings$Item, names(r_squared))]

# Add p-values to the data frame

factor_loadings$p_value <- 2 * (1 - pnorm(abs(factor_loadings$Z)))

# Rearranging the columns to desired order

factor_loadings <- factor_loadings[, c("Latente_Variabele", "Item", "Estimate", "SE", "Z", "Lambda", "R2", "p_value")]

# Print the table with factor loadings and R² values

print(factor_loadings)

# Reliability

composite_reliability <- compRelSEM(fit_ImpLevel)

print(composite_reliability)

```

| **Table E1.** Factor loadings and R2 values. | | | | | | | |
| --- | --- | --- | --- | --- | --- | --- | --- |
| **Latent Construct** | **Item** | **Estimate** | **SE** | **Z** | **Lambda** | **R2** | **p_value** |
| Client cooperation | 1 | 0.808 | 0.036 | 22.484 | 0.808 | 0.652 | 0 |
| Client cooperation | 2 | 0.810 | 0.036 | 22.539 | 0.810 | 0.656 | 0 |
| Descriptive norm | 3 | 0.852 | 0.039 | 22.091 | 0.852 | 0.725 | 0 |
| Descriptive norm | 4 | 0.869 | 0.039 | 22.336 | 0.869 | 0.756 | 0 |
| Knowledge | 1 | 0.794 | 0.033 | 24.073 | 0.794 | 0.631 | 0 |
| Knowledge | 2 | 0.850 | 0.033 | 25.565 | 0.850 | 0.723 | 0 |
| Outcome expectation | 1 | 0.894 | 0.022 | 40.056 | 0.894 | 0.799 | 0 |
| Outcome expectation | 2 | 0.851 | 0.023 | 37.269 | 0.851 | 0.724 | 0 |
| Professional obligation | 3 | 0.742 | 0.029 | 25.221 | 0.742 | 0.550 | 0 |
| Professional obligation | 1 | 0.725 | 0.030 | 24.263 | 0.725 | 0.526 | 0 |
| Professional obligation | 2 | 0.548 | 0.036 | 15.067 | 0.548 | 0.300 | 0 |
| Professional obligation | 3 | 0.596 | 0.034 | 17.315 | 0.596 | 0.355 | 0 |
| Coordinator | 1 | 0.616 | 0.047 | 13.001 | 0.616 | 0.380 | 0 |
| Coordinator | 2 | 0.853 | 0.056 | 15.259 | 0.853 | 0.728 | 0 |
| Partnership and connections | 1 | 0.867 | 0.014 | 60.002 | 0.867 | 0.752 | 0 |
| Partnership and connections | 2 | 0.808 | 0.018 | 45.383 | 0.808 | 0.653 | 0 |
| Partnership and connections | 3 | 0.853 | 0.015 | 56.089 | 0.853 | 0.728 | 0 |
| Partnership and connections | 4 | 0.632 | 0.028 | 22.715 | 0.632 | 0.400 | 0 |
| Implementation level | 1 | 0.796 | 0.028 | 28.337 | 0.796 | 0.634 | 0 |
| Implementation level | 2 | 0.790 | 0.028 | 28.064 | 0.790 | 0.625 | 0 |
| Implementation level | 3 | 0.651 | 0.031 | 21.009 | 0.651 | 0.423 | 0 |

| **Table E2.** Internal consistency. | |
| --- | --- |
| **Factor** | **Composite_Reliability** |
| Client cooperation | 0.790 |
| Descriptive norm | 0.851 |
| Knowledge | 0.808 |
| Outcome expectation | 0.865 |
| Professional obligation | 0.767 |
| Coordinator | 0.756 |
| Partnership and connections | 0.889 |
| Implementaton level | 0.795 |
