# Supplementary material for: One size fits all? A latent profile analysis to identify care professional subgroups based on implementation determinants
Source: Implement Sci Commun. 2025 Nov 17;6:121. doi: 10.1186/s43058-025-00794-x (PMC12625321; doi:10.1186/s43058-025-00794-x)
Supplement: Supplementary file 6 — Supplementary Material 6. [file 43058_2025_794_MOESM6_ESM.docx]

####### Choosing LPA model and number of classes #######

```{r choosing LPA model}

estimate_profiles(mydata, 1:10,

variances = c("equal", “equal”),

covariances = c("zero", “equal”)) %>%

compare_solutions(statistics = c("AIC", "BIC"))

LPA_many_models <- estimate_profiles(mydata, 1:10,

variances = c("equal"),

covariances = c("zero"))

# AIC and BIC values

AIC_vector_1 <- unlist(c(LPA_many_models$model_1_class_1$fit[4],

LPA_many_models$model_1_class_2$fit[4],

LPA_many_models$model_1_class_3$fit[4],

LPA_many_models$model_1_class_4$fit[4],

LPA_many_models$model_1_class_5$fit[4],

LPA_many_models$model_1_class_4$fit[4],

LPA_many_models$model_1_class_7$fit[4],

LPA_many_models$model_1_class_8$fit[4],

LPA_many_models$model_1_class_9$fit[4],

LPA_many_models$model_1_class_10$fit[4]))

BIC_vector_1 <- unlist(c(LPA_many_models$model_1_class_1$fit[6],

LPA_many_models$model_1_class_2$fit[6],

LPA_many_models$model_1_class_3$fit[6],

LPA_many_models$model_1_class_4$fit[6],

LPA_many_models$model_1_class_5$fit[6],

LPA_many_models$model_1_class_4$fit[6],

LPA_many_models$model_1_class_7$fit[6],

LPA_many_models$model_1_class_8$fit[6],

LPA_many_models$model_1_class_9$fit[6],

LPA_many_models$model_1_class_10$fit[6]))

AIC_vector_3 <- unlist(c(LPA_many_models$model_3_class_1$fit[4],

LPA_many_models$model_3_class_2$fit[4],

LPA_many_models$model_3_class_3$fit[4],

LPA_many_models$model_3_class_4$fit[4],

LPA_many_models$model_3_class_5$fit[4],

LPA_many_models$model_3_class_4$fit[4],

LPA_many_models$model_3_class_7$fit[4],

LPA_many_models$model_3_class_8$fit[4],

LPA_many_models$model_3_class_9$fit[4],

LPA_many_models$model_3_class_10$fit[4]))

BIC_vector_3 <- unlist(c(LPA_many_models$model_3_class_1$fit[6],

LPA_many_models$model_3_class_2$fit[6],

LPA_many_models$model_3_class_3$fit[6],

LPA_many_models$model_3_class_4$fit[6],

LPA_many_models$model_3_class_5$fit[6],

LPA_many_models$model_3_class_4$fit[6],

LPA_many_models$model_3_class_7$fit[6],

LPA_many_models$model_3_class_8$fit[6],

LPA_many_models$model_3_class_9$fit[6],

LPA_many_models$model_3_class_10$fit[6]))

# Create a first line for AIC and BIC model 1 profile 1:10

base::plot(AIC_vector_1, type = "b", frame = TRUE, pch = 19,

col = "red", lty = 1, ylim = c(33000,38000),

xlab="Number of profiles",

ylab="Fit indices (AIC and BIC)") +

lines(BIC_vector_1, pch = 19, col = "red", type = "b", lty = 2)

lines(AIC_vector_3, pch = 19, col = "blue", type = "b", lty = 1)

lines(BIC_vector_3, pch = 19, col = "blue", type = "b", lty = 2)

legend ("top",

legend = c("AIC model 1", "BIC model 1", "AIC model 3", "BIC model 3"),

col = c("red", "red", "blue", "blue"),

cex = 0.75,

horiz = TRUE,

bty = "n",

lty = c(1,2¸1,2),

inset=c(0, -.15), xpd=TRUE)

# Entropy values

Entropy_1 <- unlist(c(LPA_many_models$model_1_class_1$fit[12],

LPA_many_models$model_1_class_2$fit[12],

LPA_many_models$model_1_class_3$fit[12],

LPA_many_models$model_1_class_4$fit[12],

LPA_many_models$model_1_class_5$fit[12],

LPA_many_models$model_1_class_4$fit[12],

LPA_many_models$model_1_class_7$fit[12],

LPA_many_models$model_1_class_8$fit[12],

LPA_many_models$model_1_class_9$fit[12],

LPA_many_models$model_1_class_10$fit[12]))

Entropy_3 <- unlist(c(LPA_many_models$model_3_class_1$fit[12],

LPA_many_models$model_3_class_2$fit[12],

LPA_many_models$model_3_class_3$fit[12],

LPA_many_models$model_3_class_4$fit[12],

LPA_many_models$model_3_class_5$fit[12],

LPA_many_models$model_3_class_4$fit[12],

LPA_many_models$model_3_class_7$fit[12],

LPA_many_models$model_3_class_8$fit[12],

LPA_many_models$model_3_class_9$fit[12],

LPA_many_models$model_3_class_10$fit[12]))

# Create a line for Entropy model 1 profile 1:10

base::plot(Entropy_1, type = "b", frame = TRUE, pch = 19,

col = "red", lty = 1, ylim = c(0.6,1),

xlab="Number of profiles",

ylab="Entropy") +

# Add other lines for Entropy model 3

lines(Entropy_3, pch = 19, col = "blue", type = "b", lty = 1)

title("Entropy model 1 and 3") +

legend ("bottom",

col = c("red", "blue"),

cex = 0.75,

horiz = TRUE,

bty = "n",

lty = c(1),

inset=c(0, -.15), xpd=TRUE)

```

####### Saving class memberships #######

```{r save classmembership}

class_membership_1<-get_data(LPA_model_1_5)

class_membership_3<-get_data(LPA_model_3_5)

class_membership_1[,25:30]

class_membership_3[,25:30]

class_membership_1$ID<-data_zet$ID

class_membership_3$ID<-data_zet$ID

mydata_1<- left_join(mydata,class_membership_1[,25:31])

mydata_3<- left_join(mydata,class_membership_3[,25:31])

write.table(mydata_1, file = "Data_ZET_1_classes_5.csv", sep = ",", row.names = TRUE)

write.table(mydata_3, file = "Data_ZET_3_classes_5.csv", sep = ",", row.names = TRUE)

# Obtaining results class sizes

LPA_model_1_5$model_1_class_5$model$parameters$pro

LPA_model_3_5$model_3_class_5$model$parameters$pro

# Obtain class means

LPA_model_1_5$model_1_class_5$model$parameters$mean

LPA_model_3_5$model_3_class_5$model$parameters$mean

```

####### Creating a plot for model 1 with 5 profiles #######

```{r creating plot}

# Getting estimates

estimates.data_zet_1 <- get_estimates(LPA_model_1_5)

View(estimates.data_zet_1)

estimates.data_zet.del_1 <- estimates.data_zet_1[-c(23:44, 67:88, 111:132, 155:176, 199:220),]

View(estimates.data_zet.del_1)

write.table(estimates.data_zet.del_1, file = "Estimates_ZET_1_5class.csv",sep = ",", row.names = TRUE)

# alter value names

estimates.data_zet.del_1$Parameter[estimates.data_zet.del_1$Parameter == "Compatibility"] <- "Compatibility"

estimates.data_zet.del_1$Parameter[estimates.data_zet.del_1$Parameter == "Observability"] <- "Observability"

estimates.data_zet.del_1$Parameter[estimates.data_zet.del_1$Parameter == "ProceduralClarity"] <- "Procedural clarity"

estimates.data_zet.del_1$Parameter[estimates.data_zet.del_1$Parameter == "RelativePriority"] <- "Relative priority"

estimates.data_zet.del_1$Parameter[estimates.data_zet.del_1$Parameter == "AccessToKnowledge"] <- "Access to knowledge"

estimates.data_zet.del_1$Parameter[estimates.data_zet.del_1$Parameter == "Habit formation"] <- "Behavioral regulations"

estimates.data_zet.del_1$Parameter[estimates.data_zet.del_1$Parameter == "ClientCooperation_Childcheck"] <- "Client cooperation Childcheck"

estimates.data_zet.del_1$Parameter[estimates.data_zet.del_1$Parameter == "ClientCoorperation_RCCAN"] <- "Client cooperation RCCAN"

estimates.data_zet.del_1$Parameter[estimates.data_zet.del_1$Parameter == "DescriptiveNorm"] <- "Descriptive norm"

estimates.data_zet.del_1$Parameter[estimates.data_zet.del_1$Parameter == "ImplementationNeeds"] <- "Implementation needs"

estimates.data_zet.del_1$Parameter[estimates.data_zet.del_1$Parameter == "Knowledge"] <- "Knowledge"

estimates.data_zet.del_1$Parameter[estimates.data_zet.del_1$Parameter == "OutcomeExpectations""] <- "Outcome expectations"

estimates.data_zet.del_1$Parameter[estimates.data_zet.del_1$Parameter == "RelationshipClient"] <- "Relationship client"

estimates.data_zet.del_1$Parameter[estimates.data_zet.del_1$Parameter == "ProfessionalObligation"] <- "Professional obligation"

estimates.data_zet.del_1$Parameter[estimates.data_zet.del_1$Parameter == "Skills_Communication"] <- "Communication skills"

estimates.data_zet.del_1$Parameter[estimates.data_zet.del_1$Parameter == "Skills_General"] <- "General skills"

estimates.data_zet.del_1$Parameter[estimates.data_zet.del_1$Parameter == "SocialSupport"] <- "Social support"

estimates.data_zet.del_1$Parameter[estimates.data_zet.del_1$Parameter == "Coordinator"] <- "Coordinator"

estimates.data_zet.del_1$Parameter[estimates.data_zet.del_1$Parameter == "FinancialResources"] <- "Financial resources"

estimates.data_zet.del_1$Parameter[estimates.data_zet.del_1$Parameter == "FormalRatification"] <- "Formal ratification"

estimates.data_zet.del_1$Parameter[estimates.data_zet.del_1$Parameter == "PartnershipConnections"] <- "Partnership & connections"

# Adding domain variable

domain <- c("Innovation", "Innovation", "Professional", "Professional", "Professional", "Professional", "Innovation", "Innovation", "Professional", "Organization", "Organization",

"Professional", "Organization", "Professional", "Professional", "Professional", "Organization", "Professional", "Professional", "Professional", "Organization", "Professional",

"Innovation", "Innovation", "Professional", "Professional", "Professional", "Professional", "Innovation", "Innovation", "Professional", "Organization", "Organization",

"Professional", "Organization", "Professional", "Professional", "Professional", "Organization", "Professional", "Professional", "Professional", "Organization", "Professional",

"Innovation", "Innovation", "Professional", "Professional", "Professional", "Professional", "Innovation", "Innovation", "Professional", "Organization", "Organization",

"Professional", "Organization", "Professional", "Professional", "Professional", "Organization", "Professional", "Professional", "Professional", "Organization", "Professional",

"Innovation", "Innovation", "Professional", "Professional", "Professional", "Professional", "Innovation", "Innovation", "Professional", "Organization", "Organization",

"Professional", "Organization", "Professional", "Professional", "Professional", "Organization", "Professional", "Professional", "Professional", "Organization", "Professional",

"Innovation", "Innovation", "Professional", "Professional", "Professional", "Professional", "Innovation", "Innovation", "Professional", "Organization", "Organization",

"Professional", "Organization", "Professional", "Professional", "Professional", "Organization", "Professional", "Professional", "Professional", "Organization", "Professional")

estimates.data_zet.del_1$domain = domain

# Creating plot

plot_1_5C <- estimates.data_zet.del_1 %>% ggplot(aes(x=interaction(Parameter, domein, sep = "!"), Estimate, group = Class, color = factor(Class))) +

scale_fill_manual(name = "Outcomes of the Latent Profile Analysis") +

geom_point(size = 1) +

geom_line(linewidth= 0.75) +

scale_color_manual(values = c("1" = "#d395ff", "2" = "#ffaf89", "3" = "#34cd8a", "4" = "#adcdef", "5" = "#cfdc87"),

labels = c("Positive", "Integration issues", "RCCAN issues", "RCCAN and organizational issues", "Neutral")) +

ylab("Mean ratings") +

xlab("") +

labs(color = NULL) +

scale_x_discrete(guide = guide_axis_nested(delim = "!")) +

theme(axis.title.x = element_text(size=10),

axis.text.x = element_text(angle = 45, hjust = 1, size = 8),

legend.position = "top",

legend.key.size = unit(0.5, 'cm'),

legend.key.height = unit(0.5, 'cm'),

legend.key.width = unit(0.5, 'cm'),

legend.text = element_text(size=8),

axis.title.y = element_text(size=10),

axis.text.y = element_text(size=8))

```

| **Table F1.** Fit statistics for model 1 and 3 with 1 through 10 class solutions in Latent Profile Analysis. | | | | | |
| --- | --- | --- | --- | --- | --- |
| **Model** | **Classes** | **AIC** | **BIC** | **ICL** | **Entropy** |
| model 1 | 1 | 37035.22 | 37225.81 | -37225.81 | 1.00 |
| model 1 | 2 | 35625.77 | 35915.98 | -35967.16 | 0.86 |
| model 1 | 3 | 35291.90 | 35681.74 | -35744.66 | 0.89 |
| model 1 | 4 | 34804.55 | 35294.01 | -35368.12 | 0.90 |
| model 1 | 5 | 34586.97 | 35176.05 | -35254.35 | 0.91 |
| model 1 | 6 | 34452.40 | 35141.11 | -35255.30 | 0.87 |
| model 1 | 7 | 34362.04 | 35150.38 | -35260.42 | 0.89 |
| model 1 | 8 | 34249.23 | 35137.19 | -35239.41 | 0.90 |
| model 1 | 9 | 34148.62 | 35136.20 | -35255.36 | 0.89 |
| model 1 | 10 | 34132.14 | 35219.35 | -35386.84 | 0.87 |
| model 3 | 1 | 34037.59 | 35228.76 | -35228.76 | 1.000 |
| model 3 | 2 | 33808.59 | 35099.38 | -35113.63 | 0.96 |
| model 3 | 3 | 33874.02 | 35264.44 | -35322.41 | 0.88 |
| model 3 | 4 | 33785.51 | 35275.54 | -35393.99 | 0.84 |
| model 3 | 5 | 33604.74 | 35194.40 | -35277.10 | 0.90 |
| model 3 | 6 | 33681.10 | 35370.39 | -35528.39 | 0.83 |
| model 3 | 7 | 33599.44 | 35388.35 | -35554.08 | 0.83 |
| model 3 | 8 | 33555.35 | 35443.88 | -35569.27 | 0.87 |
| model 3 | 9 | 33566.79 | 35554.95 | -35732.32 | 0.84 |
| model 3 | 10 | 33552.37 | 35640.16 | -35797.08 | 0.86 |
| *Note:* AIC = [Akaike Information Criterion](https://www.sciencedirect.com/topics/psychology/akaike-information-criterion); [BIC](https://www.sciencedirect.com/topics/psychology/bayesian-information-criterion) = Bayesian Information Criterion; ICL = Integrated Completed Likelihood. | | | | | |


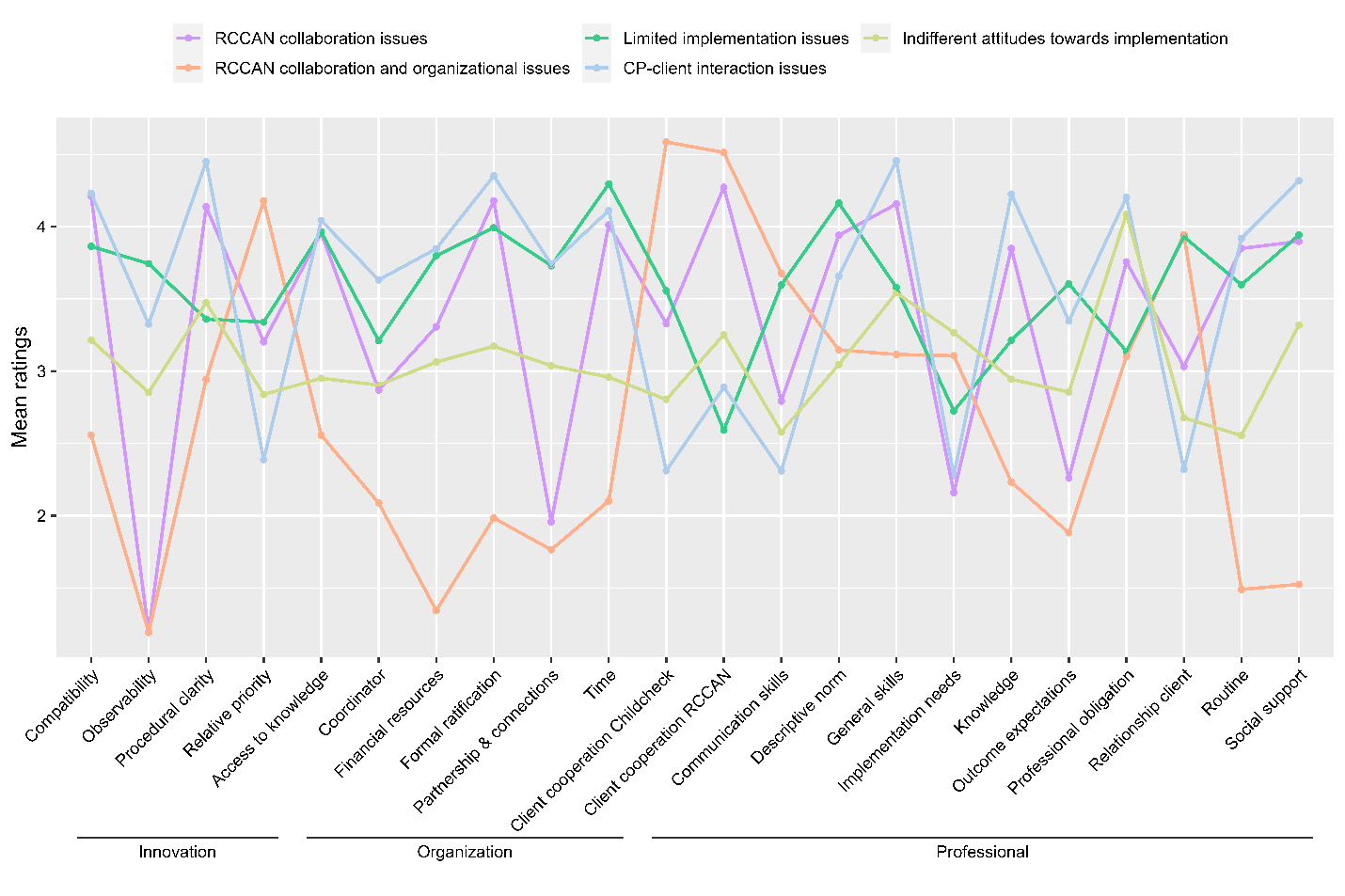


**Figure F1.** A visual representation of the five latent profiles, described across the three domains including 22 implementation determinants. The vertical axis displays mean ratings for each determinant. A higher rating means a more positive attitude concerning a specific determinant.

| **Table F2.** ANOVA results including Tukey's post-hoc comparisons for differences in determinants scores between subgroups. | | | | | | | | |
| --- | --- | --- | --- | --- | --- | --- | --- | --- |
|  | **Compatability** | | **Observability** | | **Procedural clarity** | | **Relative priority** | |
| **Comparison** | **Mean Diff** | **p_adj** | **Mean Diff** | **p_adj** | **Mean Diff** | **p_adj** | **Mean Diff** | **p_adj** |
| Overall (*F-value instead of mean difference*) | 57.922 | <0.001 | 170.302 | <0.001 | 45.63 | <0.001 | 29.135 | <0.001 |
| RCCAN collaboration and organizational issues-RCCAN collaboration issues | -1.722 | <0.001 | 0.042 | 0.999 | -1.223 | <0.001 | 0.906 | 0.001 |
| Limited implementation issues-RCCAN collaboration issues | -0.39 | 0.095 | 2.637 | <0.001 | -0.755 | <0.001 | 0.086 | 0.992 |
| CP-client interaction issues-RCCAN collaboration issues | -0.026 | 0.999 | 2.184 | <0.001 | 0.297 | 0.129 | -0.914 | <0.001 |
| Indifferent attitudes towards implementation-RCCAN collaboration issues | -1.046 | <0.001 | 1.711 | <0.001 | -0.684 | <0.001 | -0.421 | 0.036 |
| Limited implementation issues-RCCAN collaboration and organizational issues | 1.332 | <0.001 | 2.595 | <0.001 | 0.468 | 0.166 | -0.82 | 0.007 |
| CP-client interaction issues-RCCAN collaboration and organizational issues | 1.695 | <0.001 | 2.142 | <0.001 | 1.52 | <0.001 | -1.82 | <0.001 |
| Indifferent attitudes towards implementation-RCCAN collaboration and organizational issues | 0.676 | 0.001 | 1.669 | <0.001 | 0.539 | 0.024 | -1.326 | <0.001 |
| CP-client interaction issues-Limited implementation issues | 0.363 | 0.044 | -0.453 | 0.001 | 1.052 | <0.001 | -1 | <0.001 |
| Indifferent attitudes towards implementation-Limited implementation issues | -0.656 | <0.001 | -0.926 | <0.001 | 0.072 | 0.985 | -0.506 | 0.014 |
| Indifferent attitudes towards implementation-CP-client interaction issues | -1.019 | <0.001 | -0.473 | <0.001 | -0.98 | <0.001 | 0.494 | <0.001 |
|  | **Access to knowledge** | | **Behavioral regulations** | | **Client cooperation Childcheck** | | **Client cooperation RCCAN** | |
| **Comparison** | **Mean Diff** | **p_adj** | **Mean Diff** | **p_adj** | **Mean Diff** | **p_adj** | **Mean Diff** | **p_adj** |
| Overall (*F-value instead of mean difference*) | 47.662 | <0.001 | 102.224 | <0.001 | 59.028 | <0.001 | 47.725 | <0.001 |
| RCCAN collaboration and organizational issues-RCCAN collaboration issues | -1.419 | <0.001 | -2.399 | <0.001 | 1.213 | <0.001 | 0.228 | 0.820 |
| Limited implementation issues-RCCAN collaboration issues | 0.027 | 1.000 | -0.298 | 0.359 | 0.172 | 0.848 | -1.737 | <0.001 |
| CP-client interaction issues-RCCAN collaboration issues | 0.107 | 0.937 | 0.075 | 0.975 | -1.101 | <0.001 | -1.401 | <0.001 |
| Indifferent attitudes towards implementation-RCCAN collaboration issues | -1.014 | <0.001 | -1.312 | <0.001 | -0.581 | <0.001 | -1.064 | <0.001 |
| Limited implementation issues-RCCAN collaboration and organizational issues | 1.445 | <0.001 | 2.102 | <0.001 | -1.041 | <0.001 | -1.965 | <0.001 |
| CP-client interaction issues-RCCAN collaboration and organizational issues | 1.526 | <0.001 | 2.474 | <0.001 | -2.315 | <0.001 | -1.629 | <0.001 |
| Indifferent attitudes towards implementation-RCCAN collaboration and organizational issues | 0.405 | 0.236 | 1.087 | <0.001 | -1.794 | <0.001 | -1.292 | <0.001 |
| CP-client interaction issues-Limited implementation issues | 0.08 | 0.983 | 0.373 | 0.048 | -1.274 | <0.001 | 0.336 | 0.140 |
| Indifferent attitudes towards implementation-Limited implementation issues | -1.04 | <0.001 | -1.014 | <0.001 | -0.753 | <0.001 | 0.673 | <0.001 |
| Indifferent attitudes towards implementation-CP-client interaction issues | -1.12 | <0.001 | -1.387 | <0.001 | 0.52 | <0.001 | 0.337 | 0.003 |

|  | **Descriptive norm** | | **Implementation needs** | | **Knowledge** | | **Outcome expectations** | |
| --- | --- | --- | --- | --- | --- | --- | --- | --- |
| **Comparison** | **Mean Diff** | **p_adj** | **Mean Diff** | **p_adj** | **Mean Diff** | **p_adj** | **Mean Diff** | **p_adj** |
| Overall (*F-value instead of mean difference*) | 31.794 | <0.001 | 26.043 | <0.001 | 90.14 | <0.001 | 35.298 | <0.001 |
| RCCAN collaboration and organizational issues-RCCAN collaboration issues | -0.79 | <0.001 | 0.961 | 0.001 | -1.625 | <0.001 | -0.388 | 0.328 |
| Limited implementation issues-RCCAN collaboration issues | 0.245 | 0.506 | 0.554 | 0.056 | -0.686 | <0.001 | 1.36 | <0.001 |
| CP-client interaction issues-RCCAN collaboration issues | -0.279 | 0.127 | 0.073 | 0.990 | 0.401 | <0.001 | 1.061 | <0.001 |
| Indifferent attitudes towards implementation-RCCAN collaboration issues | -0.898 | <0.001 | 1.079 | <0.001 | -0.9 | <0.001 | 0.597 | <0.001 |
| Limited implementation issues-RCCAN collaboration and organizational issues | 1.035 | <0.001 | -0.407 | 0.521 | 0.938 | <0.001 | 1.748 | <0.001 |
| CP-client interaction issues-RCCAN collaboration and organizational issues | 0.511 | 0.021 | -0.888 | 0.001 | 2.026 | <0.001 | 1.449 | <0.001 |
| Indifferent attitudes towards implementation-RCCAN collaboration and organizational issues | -0.108 | 0.968 | 0.118 | 0.985 | 0.724 | <0.001 | 0.985 | <0.001 |
| CP-client interaction issues-Limited implementation issues | -0.524 | 0.001 | -0.481 | 0.041 | 1.087 | <0.001 | -0.299 | 0.210 |
| Indifferent attitudes towards implementation-Limited implementation issues | -1.143 | <0.001 | 0.525 | 0.020 | -0.214 | 0.434 | -0.762 | <0.001 |
| Indifferent attitudes towards implementation-CP-client interaction issues | -0.619 | <0.001 | 1.006 | <0.001 | -1.301 | <0.001 | -0.464 | <0.001 |
|  | **Relationship client** | | **Professional obligation** | | **Communication skills** | | **General skills** | |
| **Comparison** | **Mean Diff** | **p_adj** | **Mean Diff** | **p_adj** | **Mean Diff** | **p_adj** | **Mean Diff** | **p_adj** |
| Overall (*F-value instead of mean difference*) | 49.702 | <0.001 | 31.312 | <0.001 | 30.23 | <0.001 | 40.719 | <0.001 |
| RCCAN collaboration and organizational issues-RCCAN collaboration issues | 0.873 | <0.001 | -0.681 | 0.001 | 0.926 | <0.001 | -1.044 | <0.001 |
| Limited implementation issues-RCCAN collaboration issues | 0.909 | <0.001 | -0.608 | <0.001 | 0.816 | <0.001 | -0.623 | 0.001 |
| CP-client interaction issues-RCCAN collaboration issues | -0.78 | <0.001 | 0.431 | 0.001 | -0.477 | 0.003 | 0.32 | 0.069 |
| Indifferent attitudes towards implementation-RCCAN collaboration issues | -0.431 | 0.011 | 0.34 | 0.017 | -0.207 | 0.528 | -0.605 | <0.001 |
| Limited implementation issues-RCCAN collaboration and organizational issues | 0.036 | 1.000 | 0.073 | 0.994 | -0.111 | 0.987 | 0.421 | 0.231 |
| CP-client interaction issues-RCCAN collaboration and organizational issues | -1.653 | <0.001 | 1.112 | <0.001 | -1.403 | <0.001 | 1.365 | <0.001 |
| Indifferent attitudes towards implementation-RCCAN collaboration and organizational issues | -1.304 | <0.001 | 1.021 | <0.001 | -1.133 | <0.001 | 0.44 | 0.089 |
| CP-client interaction issues-Limited implementation issues | -1.689 | <0.001 | 1.039 | <0.001 | -1.292 | <0.001 | 0.943 | <0.001 |
| Indifferent attitudes towards implementation-Limited implementation issues | -1.34 | <0.001 | 0.948 | <0.001 | -1.022 | <0.001 | 0.018 | 1.000 |
| Indifferent attitudes towards implementation-CP-client interaction issues | 0.349 | 0.002 | -0.091 | 0.746 | 0.27 | 0.029 | -0.925 | <0.001 |

|  | **Social support** | | **Coordinator** | | **Financial resources** | | **Formal ratification** | |
| --- | --- | --- | --- | --- | --- | --- | --- | --- |
| **Comparison** | **Mean Diff** | **p_adj** | **Mean Diff** | **p_adj** | **Mean Diff** | **p_adj** | **Mean Diff** | **p_adj** |
| Overall (*F-value instead of mean difference*) | 66.272 | <0.001 | 20.017 | <0.001 | 36.015 | <0.001 | 71.434 | <0.001 |
| RCCAN collaboration and organizational issues-RCCAN collaboration issues | -2.364 | <0.001 | -0.782 | 0.012 | -2.032 | <0.001 | -2.233 | <0.001 |
| Limited implementation issues-RCCAN collaboration issues | 0.061 | 0.997 | 0.363 | 0.359 | 0.531 | 0.106 | -0.197 | 0.785 |
| CP-client interaction issues-RCCAN collaboration issues | 0.471 | 0.006 | 0.735 | <0.001 | 0.521 | 0.015 | 0.157 | 0.759 |
| Indifferent attitudes towards implementation-RCCAN collaboration issues | -0.543 | 0.001 | 0.041 | 0.999 | -0.259 | 0.530 | -1.025 | <0.001 |
| Limited implementation issues-RCCAN collaboration and organizational issues | 2.425 | <0.001 | 1.145 | <0.001 | 2.563 | <0.001 | 2.036 | <0.001 |
| CP-client interaction issues-RCCAN collaboration and organizational issues | 2.835 | <0.001 | 1.517 | <0.001 | 2.554 | <0.001 | 2.389 | <0.001 |
| Indifferent attitudes towards implementation-RCCAN collaboration and organizational issues | 1.82 | <0.001 | 0.823 | 0.002 | 1.773 | <0.001 | 1.208 | <0.001 |
| CP-client interaction issues-Limited implementation issues | 0.41 | 0.051 | 0.373 | 0.161 | -0.009 | 1.000 | 0.354 | 0.102 |
| Indifferent attitudes towards implementation-Limited implementation issues | -0.604 | 0.001 | -0.322 | 0.298 | -0.79 | <0.001 | -0.828 | <0.001 |
| Indifferent attitudes towards implementation-CP-client interaction issues | -1.015 | <0.001 | -0.695 | <0.001 | -0.781 | <0.001 | -1.181 | <0.001 |
|  | **Partnership and connections** | | **Time** | |  |  |  |  |
| **Comparison** | **Mean Diff** | **p_adj** | **Mean Diff** | **p_adj** |  |  |  |  |
| Overall (*F-value instead of mean difference*) | 94.892 | <0.001 | 70.76 | <0.001 |  |  |  |  |
| RCCAN collaboration and organizational issues-RCCAN collaboration issues | -0.172 | 0.877 | -1.929 | <0.001 |  |  |  |  |
| Limited implementation issues-RCCAN collaboration issues | 1.807 | <0.001 | 0.321 | 0.339 |  |  |  |  |
| CP-client interaction issues-RCCAN collaboration issues | 1.797 | <0.001 | 0.127 | 0.870 |  |  |  |  |
| Indifferent attitudes towards implementation-RCCAN collaboration issues | 1.112 | <0.001 | -1.044 | <0.001 |  |  |  |  |
| Limited implementation issues-RCCAN collaboration and organizational issues | 1.979 | <0.001 | 2.249 | <0.001 |  |  |  |  |
| CP-client interaction issues-RCCAN collaboration and organizational issues | 1.969 | <0.001 | 2.056 | <0.001 |  |  |  |  |
| Indifferent attitudes towards implementation-RCCAN collaboration and organizational issues | 1.284 | <0.001 | 0.884 | <0.001 |  |  |  |  |
| CP-client interaction issues-Limited implementation issues | -0.01 | 1.000 | -0.193 | 0.661 |  |  |  |  |
| Indifferent attitudes towards implementation-Limited implementation issues | -0.695 | <0.001 | -1.365 | <0.001 |  |  |  |  |
| Indifferent attitudes towards implementation-CP-client interaction issues | -0.685 | <0.001 | -1.172 | <0.001 |  |  |  |  |
